# Supplementary material for: Closed-loop recycling of sulfur-rich polymers with tunable properties spanning thermoplastics, elastomers, and vitrimers
Source: Nat Commun. 2024 Apr 8;15:3002. doi: 10.1038/s41467-024-47382-6 (PMC11001992; doi:10.1038/s41467-024-47382-6)
Supplement: Supplementary file 1 — Supplementary Information [file 41467_2024_47382_MOESM1_ESM.pdf]

Supplementary Information

Closed-Loop Recycling of Sulfur-Rich Polymers with Tunable Properties Spanning Thermoplastics, Elastomers, and Vitrimers

*Jin-Zhuo Zhao, Tian-Jun Yue, Bai-Hao Ren, Xiao-Bing Lu, and Wei-Min Ren**

[1]State Key Laboratory of Fine Chemicals, Frontiers Science Center for Smart Materials, Dalian University of Technology, 2 Linggong Road, Dalian, 116024, China.

*E-mail: wmren@dlut.edu.cn.

Corresponding Author

***Wei-Min Ren** – State Key Laboratory of Fine Chemicals, Frontiers Science Center for Smart Materials, Dalian University of Technology, 2 Linggong Road, Dalian, 116024, China; orcid.org/0000-0003-4425-1453; E-mail: wmren@dlut.edu.cn.

Contents

1. Synthesis of dimethyl trithiocarbonate and dithiols.

2. General procedure for the polycondensation of dithiols and dimethyl trithiocarbonate

3. Thermal properties of polytrithiocarbonates: **P1**–**P7**

4. Crystallinity values of polytrithiocarbonates: **P1**–**P7**

5. Thermal properties of polytrithiocarbonates: **P8**–**P11**

6. Dynamic mechanical properties of polytrithiocarbonates: **P1**–**P10**

7. Synthesis of polytrithiocarbonate-based thermoplastic elastomer

8. Thermal properties and dynamic mechanical properties of copolymers: **P12**–**P14**

9. ^{1}H NMR spectra of polytrithiocarbonates containing disulfide bonds

10. Synthesis of vitrimer **P15**

11. Closed-loop recycling of polytrithiocarbonates

12. NMR spectra of DMTC, dithiols and polytrithiocarbonates

13. GPC traces of **P1**–**P7**

14. Reference

1.Synthesis of dimethyl trithiocarbonate and dithiols

1.1 General procedure for the synthesis of dimethyl trithiocarbonate (DMTC)

Synthesis of DMTC.

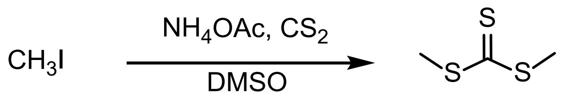

The dimethyl trithiocarbonate (DMTC) was synthesized according to the literature[1] after minor optimization as follows: To a solution of NH_4OAc (40 g, 520 mmol, 2.6 equiv.) in dimethyl sulfoxide (160 mL), CS_2 (14.4 g, 240 mmol, 1.2 equiv.) was added, followed by the vigorous stirring at room temperature for 15 min. The color of the solution turned into dark red. Then, Iodomethane (28.4 g, 200 mmol, 1.0 equiv.) was added, followed by stirring for 8 h at room temperature where the color of the solution gradually fades. Dichloromethane (240 mL) was added to quench the reaction. The reaction mixture was washed by water (800 mL × 2). The organic phase was collected and dried over Na_2SO_4 for 4 h, followed by removing out volatile via rotary evaporated under vacuum. The residue was further purified by column chromatography (PE/DCM=100/1) to afford pure DMTC as an orange oil (15.2 g, yield 66%). ^{1}H NMR (400 MHz, $CDCl_3$) δ 2.76 (s, 1H). ^{13}C NMR (100 MHz, $CDCl_3$) δ 225.50 (d, J = 2.1 Hz), 20.05.

1.2 General procedure for the synthesis of dithiols

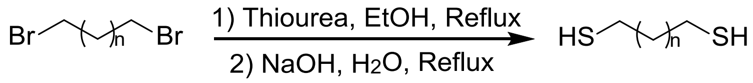

Synthesis of 1,12-dodecanedithiol

The 1,12-dodecanedithiol was synthesized according to the literature[2] after minor optimization as follows: A mixture of 1,12-dibromododecane (45 mmol, 14.7 g, 1 equiv.), thiourea (95 mmol 7.2 g, 2.1 equiv.), and 400 mL of ethanol was refluxed under N_2 atmosphere for 18 h. Then, the solution of sodium hydroxide (250 mmol, 10 g, 5.5 equiv.) in 300 mL of water was added. The resulting mixture was further refluxed under N_2 atmosphere for 4 h. After cooling to room temperature, the solution was extracted with diethyl ether (350 mL × 2), and combined organic extracts were dried over anhydrous $MgSO_4$ for 4 h. The solvent was removed under the vacuum, and the crude product was recrystallized twice from methanol to yield a white solid (39.6 mmol, 9.3g yield 89%). ^{1}H NMR (400 MHz, $CDCl_3$) δ 2.52 (q, J = 7.4 Hz, 4H), 1.61 (p, J = 7.2 Hz, 4H), 1.38 (d, J = 7.8 Hz, 4H), 1.33 (t, J = 7.7 Hz, 2H), 1.25 (s,12H). ^{13}C NMR (10 MHz, $CDCl_3$) δ 34.05, 29.52 (d, J = 4.6 Hz), 29.07, 28.38, 24.66.

Synthesis of 1,16-hexadecanedithiol

1,16-hexadecanedithiol was prepared in a similar method as 1,12-dodecanedithiol. (38.3 mmol, 11.1 g, white solid, yield 85%).^{1}H NMR (400 MHz, $CDCl_3$) δ 2.52 (q, J = 7.4 Hz, 4H), 1.61 (p, J = 7.3 Hz, 4H), 1.38 (q, J = 7.4 Hz, 4H), 1.33 (t, J = 7.8 Hz, 2H), 1.26 (d, J = 6.6 Hz, 20H). ^{13}C NMR (100 MHz, $CDCl_3$) δ 34.07, 30.01 – 29.37 (m), 29.09, 28.40, 24.68.

Synthesis of 1,18-octadecanedithiol

1,18-octadecanedithiol was prepared in a similar method as 1,12-dodecanedithiol. (39.2 mmol, 12.5 g, white solid, yield 87%).^{1}H NMR (400 MHz, $CDCl_3$) δ 2.52 (q, J = 7.4 Hz, 4H), 1.61 (p, J = 7.3 Hz, 4H), 1.37 (t, J = 7.5 Hz, 4H), 1.33 (t, J = 7.7 Hz, 2H), 1.26 (d, J = 6.9 Hz, 24H). ^{13}C NMR (100 MHz, $CDCl_3$) δ 34.07, 30.29 – 29.33 (m), 29.09, 28.40, 24.68.

2 General procedure for the polycondensation of dithiols and dimethyl trithiocarbonate.

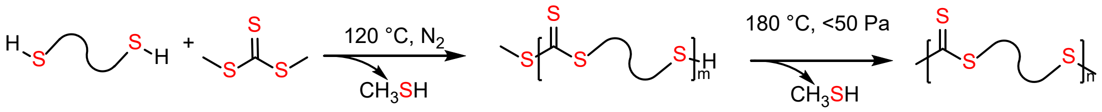

In a typical polycondensation procedure: Dithiol (50 mmol, 1.0 equiv.) and DMTC (50 mmol, 1.0 equiv.) were added to the three-necked flask (~50 mL) of the polycondensation device, which is consisted by a three-necked flask (~50 mL) equipped with a mechanical stirrer, distillation apparatus, and a manifold equipped with vacuum and N_2 gas lines. After degassing by bubbling with N_2 for 30 min, KH (0.25 mmol, 0.005 equiv.) was added and the flask was immersed in a hot-oil bath (120 °C). The reaction was started and continued for 2.0 h with the pressure gradually reduced to 70000 Pa, followed by gradually reducing the pressure to 2000 Pa, accompanied with the bath temperature improving to 150 °C and maintaining for 2.0 h. Then, the bath temperature was further elevated to 180 °C and maintained at 180 °C, wherein the pressure of reaction system was reduced to 50 Pa. The polycondensation procedure was finished after the Weissenberg phenomenon appeared for 2.0 h. The polymer melt was diluted with xylene at 120 °C followed by pouring the solution into large volume of MeOH to give the polymer precipitated, which was washed with MeOH and dried in a vacuum oven at 50 °C to afford polytrithiocarbonate.

Supplementary Table 1 The polycondensation of 1,10-decanedithiol with dimethyl trithiocarbonate.[a]

Entry	Cat.	[Dithiol]/[DMTC]/[Cat.]	Temp.[b] (°C)	Conv.[c] (%)	Time[d] (h)	Yield[e] (%)	M_n[f] (kg/mol)	$Đ$[f]
1	LiH	200/200/1	40	1	12[g]	30	3.4	2.92
2	NaH	200/200/1	40	2	12[g]	60	3.9	3.18
3	KH	200/200/1	40	4	12[g]	70	9.1	2.63
4	KH	200/200/1	80	7	12[g]	85	16.0	2.31
5	KH	200/200/1	120	50	10	95	44.0	2.55
6	KH	100/100/1	120	50	9	98	41.9	2.47
7	KH	1000/1000/1	120	47	15	98	39.4	2.63

[a] Polycondensation was conducted in a two-steps procedure: the first step was transesterification under 70000 Pa for 2.0 h; the second step was polycondensation at 150 °C, 2000 Pa for 2.0 h, and then 180 °C, 50 Pa until the viscosity of the system no longer changes. [b] Reaction temperature of transesterification process. [c] The conversion of $-CH_2SH$ was determined by the signals observed in the ^1H NMR spectra of the oligomers generated in transesterification. [d] The time the reaction terminated when the viscosity of the system no longer changes. [e] The yield calculated by the precipitated polymers. [f] Molecular weight and molecular weight distribution of the polymers with low solubility at room temperature were determined by gel permeation chromatography (GPC) at 150 °C using 1,2,4-trichlorobenzene as the eluent and calibrated with polystyrene standards. [g] n.d. = not determined.

3.Thermal properties of polytrithiocarbonates: **P1–P11**

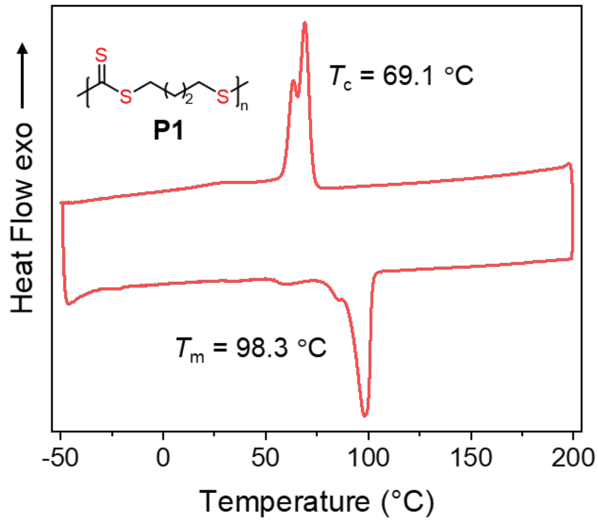

Supplementary Fig. 1 DSC thermogram of **P1** with T_m = 98.3 °C and T_c = 69.1 °C.

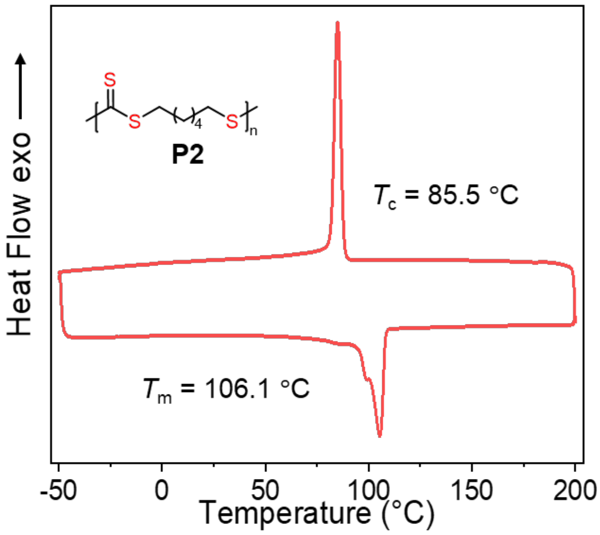

Supplementary Fig. 2 DSC thermogram of **P2** with T_m = 106.1 °C and T_c = 85.5 °C.

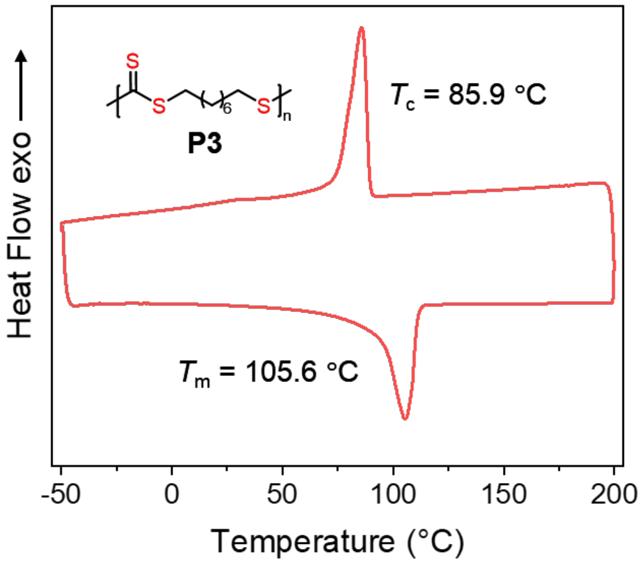

Supplementary Fig. 3 DSC thermogram of **P3** with T_m = 105.6 °C and T_c = 85.9 °C.

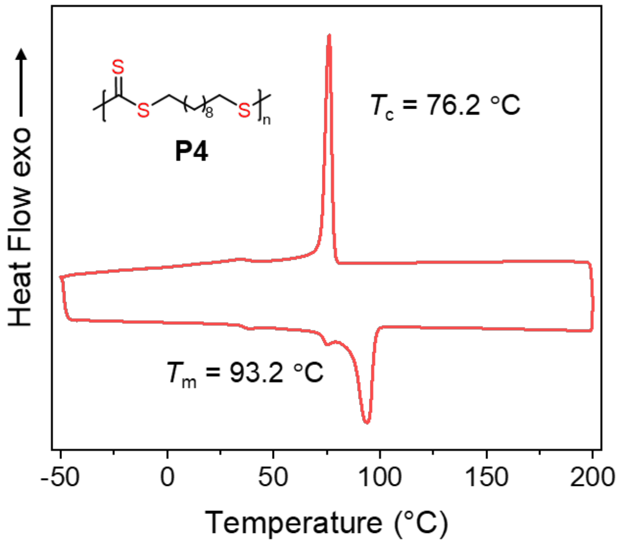

Supplementary Fig. 4 DSC thermogram of **P4** with T_m = 93.2 °C and T_c = 76.2 °C.

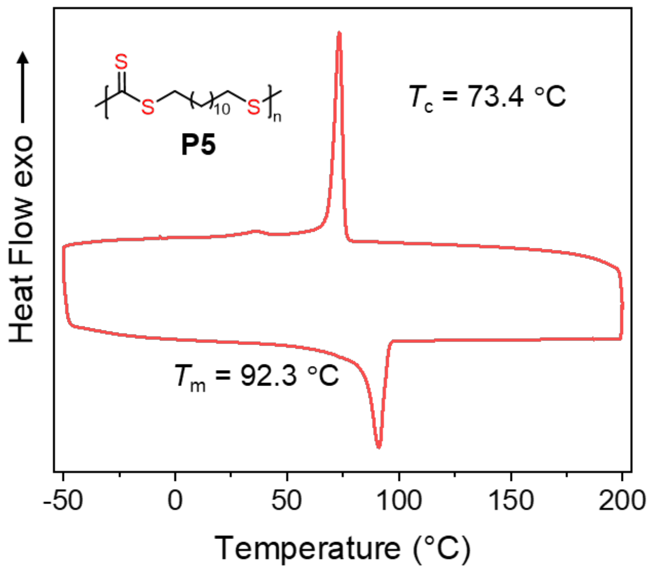

Supplementary Fig. 5 DSC thermogram of **P5** with T_m = 92.3 °C and T_c = 73.4 °C.

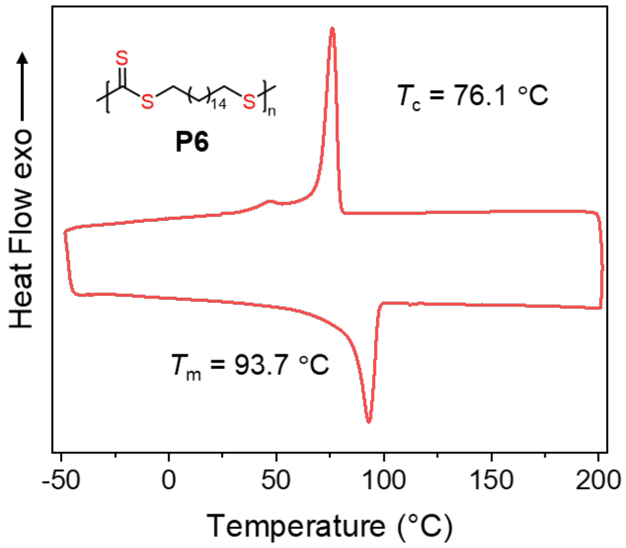

Supplementary Fig. 6 DSC thermogram of **P6** with T_m = 93.7 °C and T_c = 76.1 °C.

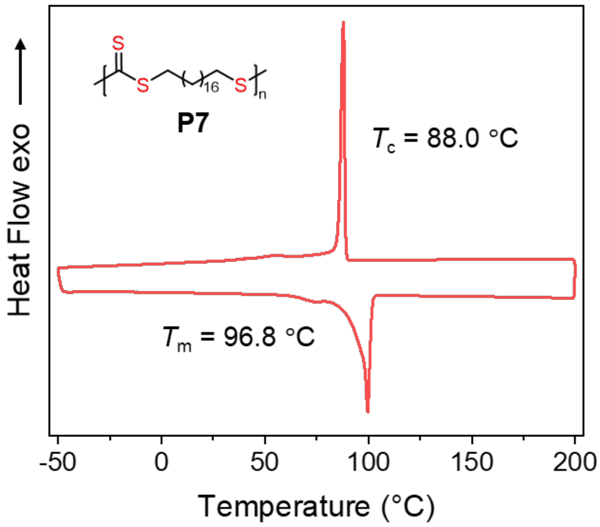

Supplementary Fig. 7 DSC thermogram of **P7** with T_m = 96.8 °C and T_c = 88.0 °C.

4. Crystallinity values of polytrithiocarbonates: P1–P7

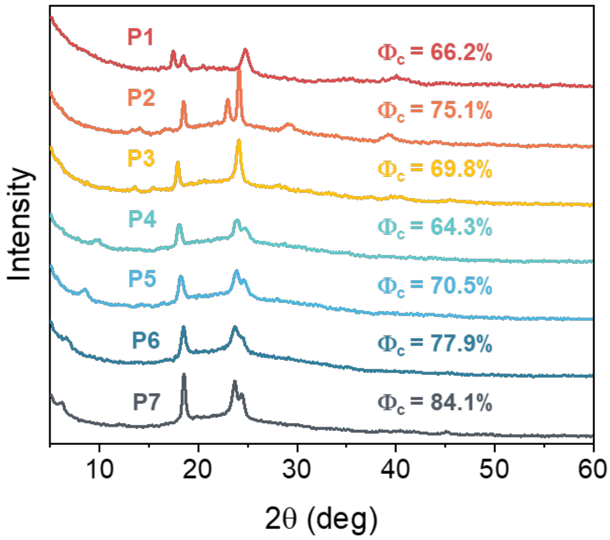

Supplementary Fig. 8 Powder XRD profiles of **P1**−**P7**. The Crystallinity values (Φc) of **P1**−**P7** determined by deconvolution of WAXS diffractograms were indicated.

5. Thermal properties of polytrithiocarbonates: P8–P11

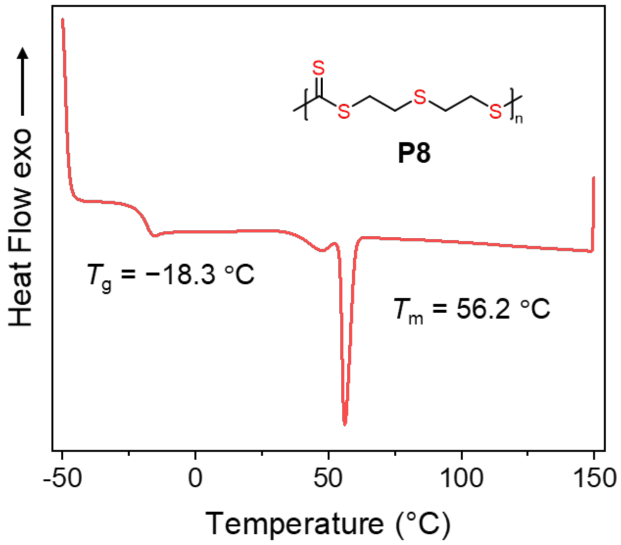

Supplementary Fig. 9 DSC thermogram of **P8** with $T_g = -18.3$ °C and $T_m = 56.2$ °C.

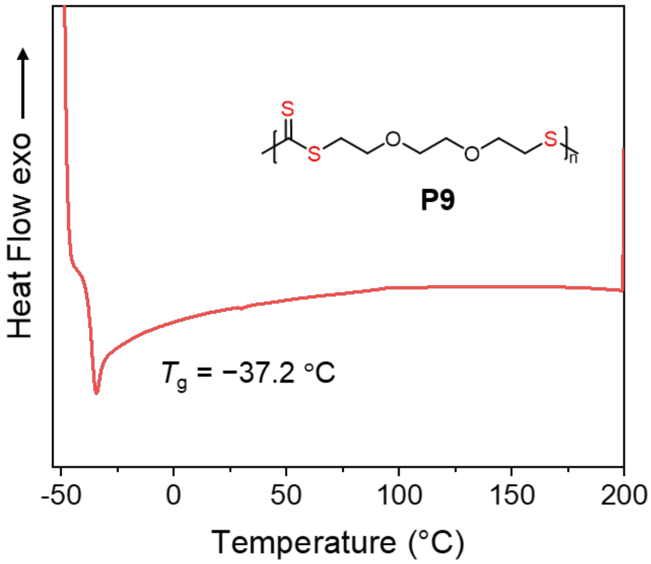

Supplementary Fig. 10 DSC thermogram of **P9** with $T_g = -37.2$ °C.

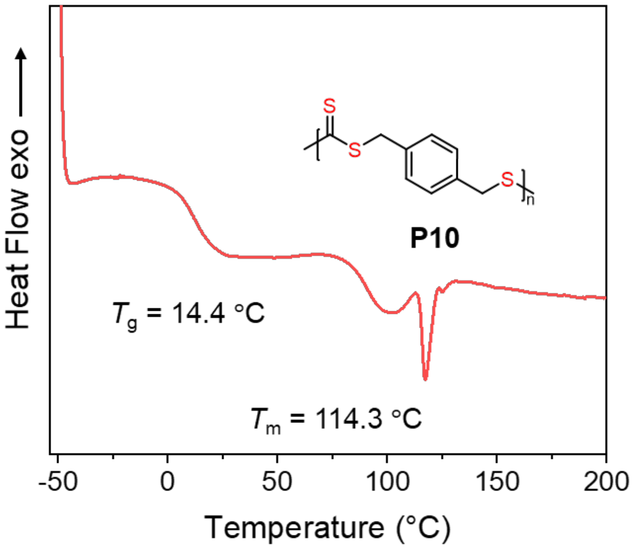

Supplementary Fig. 11 DSC thermogram of **P10** with T_g = 14.4 °C and T_m = 114.3 °C.

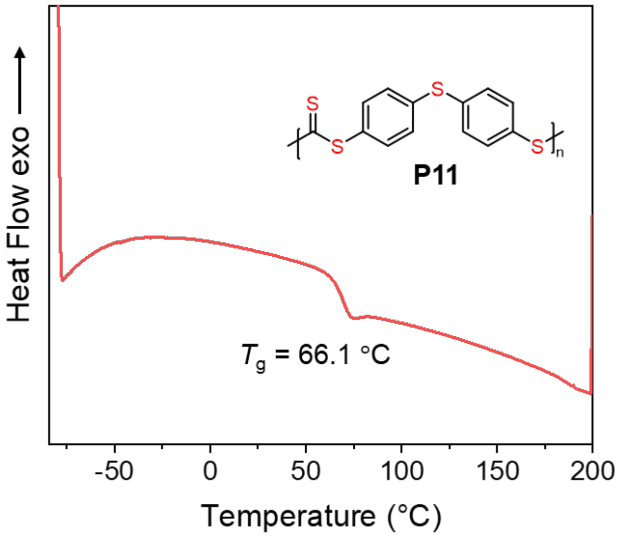

Supplementary Fig. 12 DSC thermogram of **P11** with T_g = 66.1 °C.

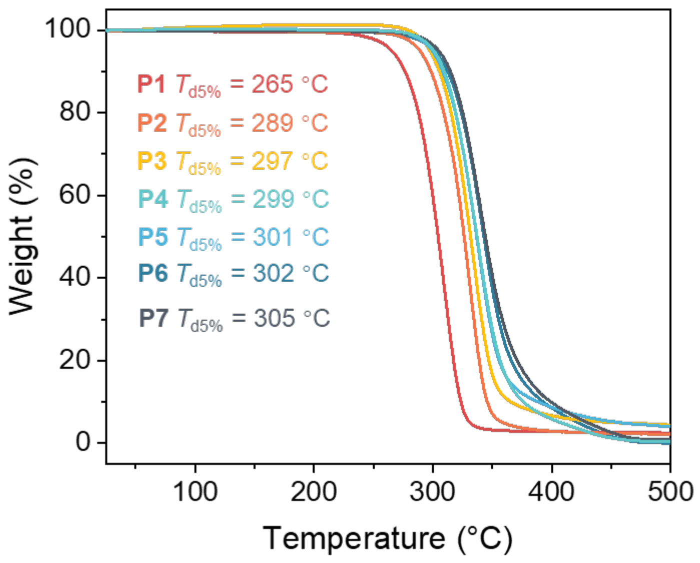

Supplementary Fig. 13 TGA thermograms of **P1−P7**.

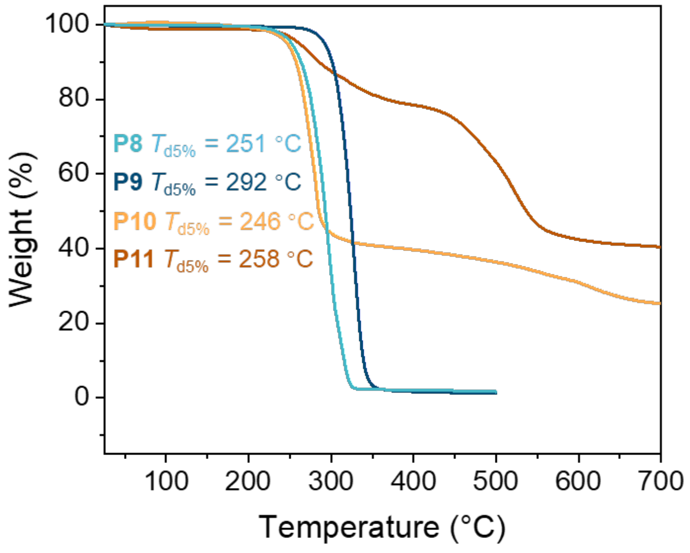

Supplementary Fig. 14 TGA thermograms of **P8−P11**.

6. Dynamic mechanical properties of polytrithiocarbonates: P1–P10

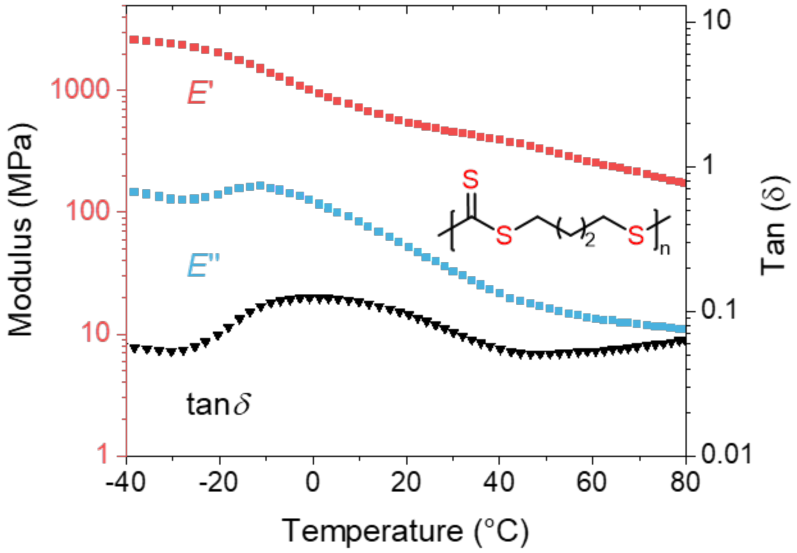

Supplementary Fig. 15 Overlay of storage modulus E', loss modulus E'', and tanδ for **P1** characterized by DMA.

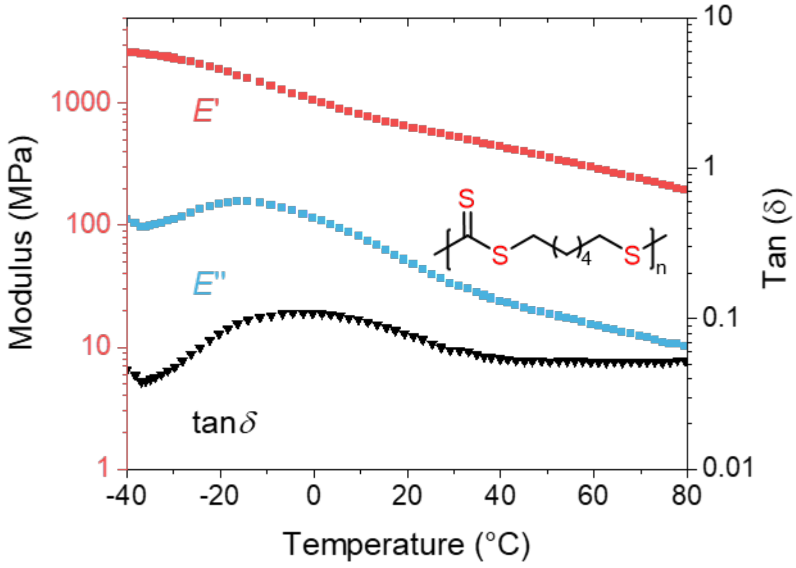

Supplementary Fig. 16 Overlay of storage modulus E', loss modulus E'', and tanδ for **P2** characterized by DMA.

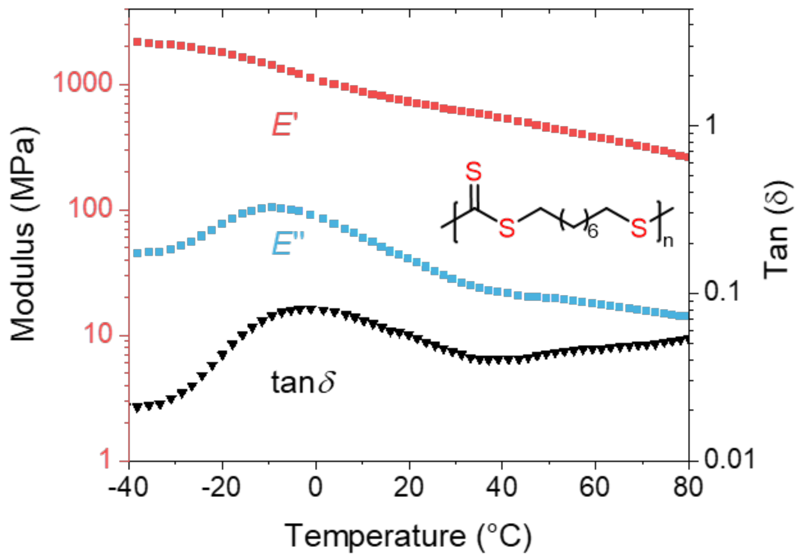

Supplementary Fig. 17 Overlay of storage modulus E', loss modulus E'', and tanδ for **P3** characterized by DMA.

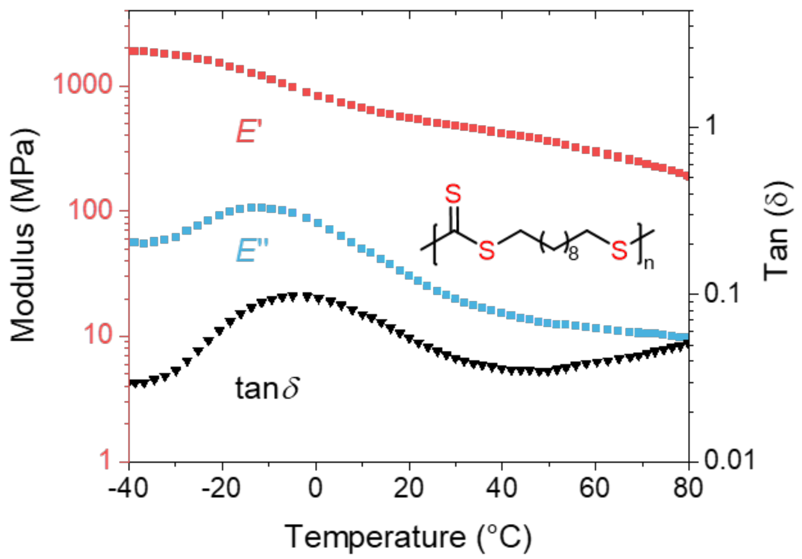

Supplementary Fig. 18 Overlay of storage modulus E', loss modulus E'', and tanδ for **P4** characterized by DMA.

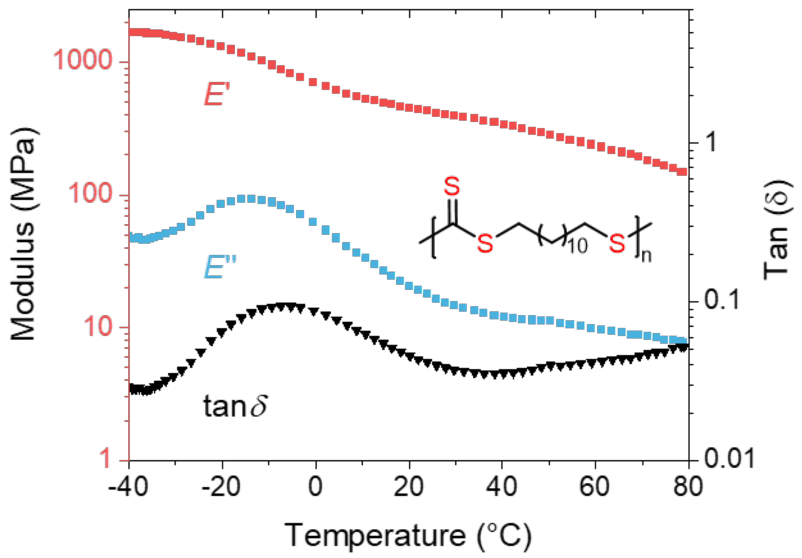

Supplementary Fig. 19 Overlay of storage modulus E', loss modulus E'', and tanδ for **P5** characterized by DMA.

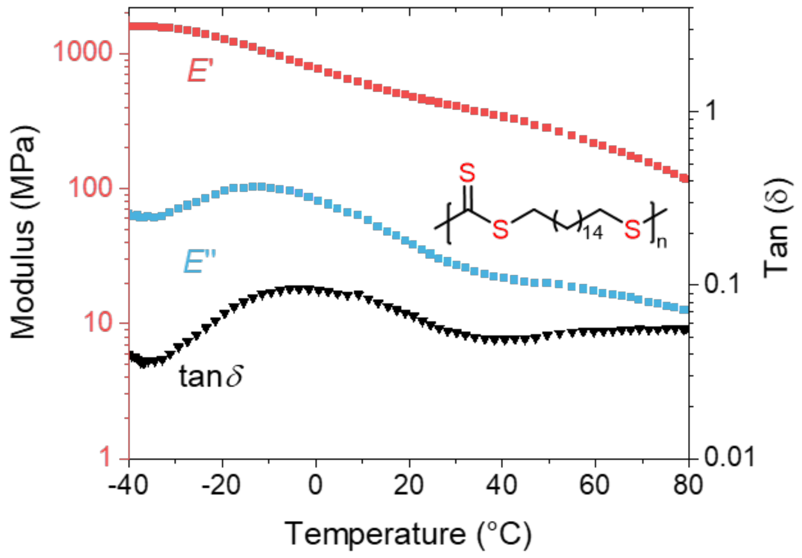

Supplementary Fig. 20 Overlay of storage modulus E', loss modulus E'', and tanδ for **P6** characterized by DMA.

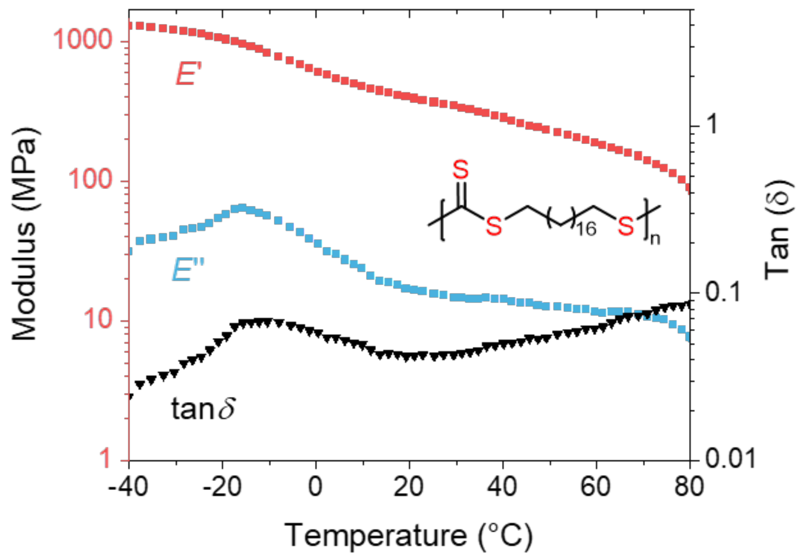

Supplementary Fig. 21 Overlay of storage modulus E', loss modulus E'', and tanδ for **P7** characterized by DMA.

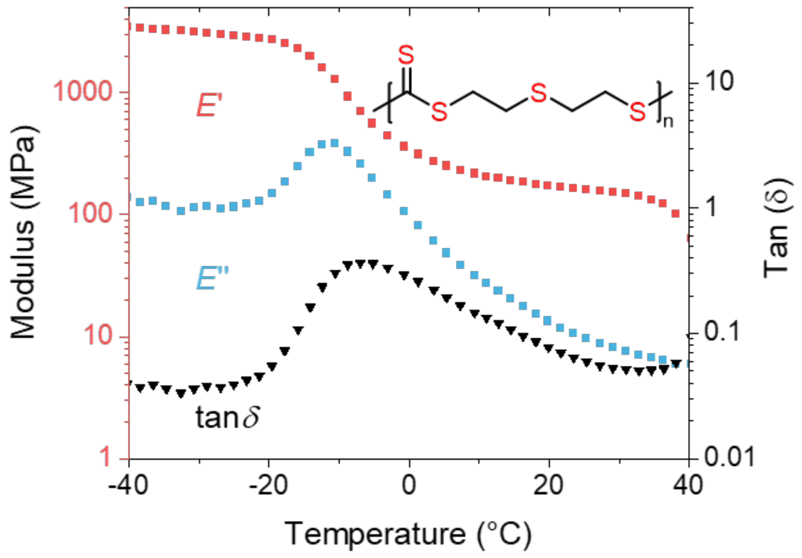

Supplementary Fig. 22 Overlay of storage modulus E', loss modulus E'', and tanδ for **P8** characterized by DMA.

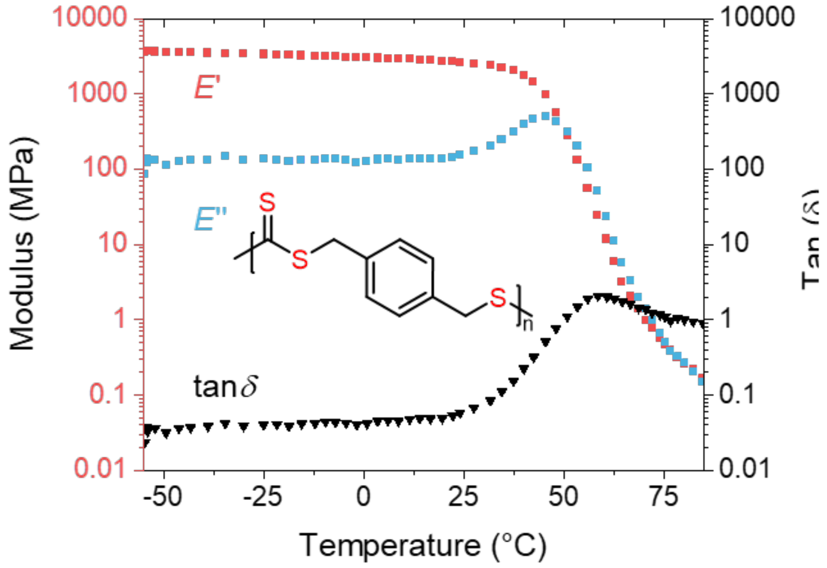

Supplementary Fig. 23 Overlay of storage modulus *E′*, loss modulus *E″*, and tan*δ* for **P10** characterized by DMA.

7. Synthesis of polytrithiocarbonate-based thermoplastic elastomer

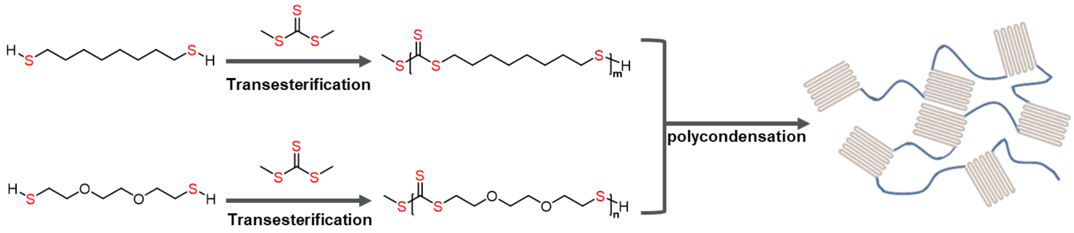

Representative procedure for the synthesis of copolymers is given with copolymer **P12** as example:

3 (37.5 mmol, 6.7 g, 3 equiv.), together with DMTC (37.5 mmol, 5.2 g, 3 equiv.) and **9** (12.5 mmol, 2.3 g, 1 equiv.), together with DMTC (12.5 mmol, 1.7 g, 1 equiv.) were added to two sets of polycondensation devices, respectively. After degassing through N_2 bubbling for 30 min, KH (0.5% equiv.) was added to these two reaction systems, respectively, followed by immersing the flasks into the hot-oil bath (120 °C), respectively. The reaction was conducted for 2.0 h with the pressure gradually reduced to70000 Pa. After switching the reaction system to a nitrogen atmosphere, **P9** oligomer was transferred to the reaction system of synthesizing **P3** oligomer. Then, the polycondensation was continued as the representative polycondensation procedures described above. **P13** and **P14** were synthesized in the same procedure with different feed ratio of **3** to **9**, as shown in Supplementary Table 2.

Supplementary Table 2 The copolymerization of **3** and **9**.[a]

Entry	Polymer	[**3**]/[**9**]/[Cat.]	M_n[b] (kg/mol)	$Đ$[b]	T_g[c] (°C)	T_m[c] (°C)	T_c[c] (°C)	$T_{d5\%}$[d] (°C)	σ_B[e] (MPa)	ε_B[e] (%)	E'[f] (MPa)	E[g] (MPa)	Yield[f] (%)
1	**P12**	150/50/1	50.2	2.00	−46.0	78.7	52.8	290	14.3	320	122	182	98
2	**P13**	100/100/1	48.3	2.01	−45.7	38.1	−8.6	261	6.6	332	24	13	98
3	**P14**	50/150/1	42.6	2.02	−45.9	n.d.	n.d.	251	n.d. [h]	n.d.	n.d.	n.d.	96

[a] Polymerization performed at 120 °C and 70000 Pa for 2.0 h, followed by the polycondensation at 150 °C and 2000 Pa for 2.0 h, then 180 °C and 50 Pa until 2.0 h after the Weissenberg phenomenon. [b] Molecular weight and molecular weight distribution of the polymers with low solubility at room temperature were determined by gel permeation chromatography (GPC) equipped with a triple detection array, including a differential refractive index (RI) detector, a two-angle light scattering (LS) detector, and a fourbridge capillary viscometer at 150 °C using 1,2,4-trichlorobenzene as the solvent. [c] Glass transition temperature (T_g), melting temperature (T_m) and crystalline temperature (T_c) were determined using DSC. [d] Temperature at a molecular weight loss of 5% determined using TGA. [e] Breaking strength (σ_B) and elongation at break (ε_B) determined by Uniaxial tensile elongation testing. [f] Storage modulus at 25 °C determined using DMA. [g] Young's modulus (E) calculated as the slope from 0 to 1% strain. [h] Not determined. [f] The yield calculated by the precipitated polymers.

8. Thermal properties and dynamic mechanical properties of copolymers: P12–P14

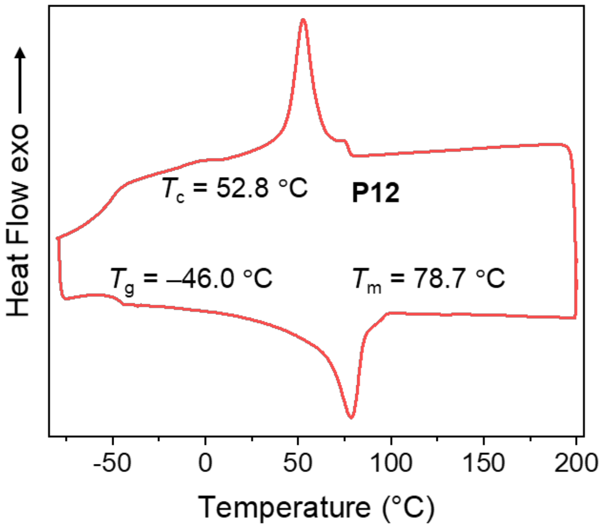

Supplementary Fig. 24 DSC thermogram of **P12** with T_g = –46.0 °C, T_m = 78.7 °C and T_c = 52.8 °C.

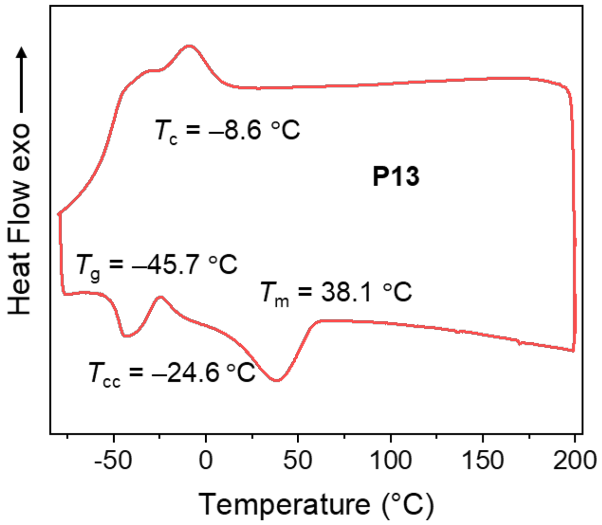

Supplementary Fig. 25 DSC thermogram of **P13** with T_g = –45.7 °C, T_{cc} = –24.7 °C, T_m = 38.1 °C and T_c = –8.6 °C.

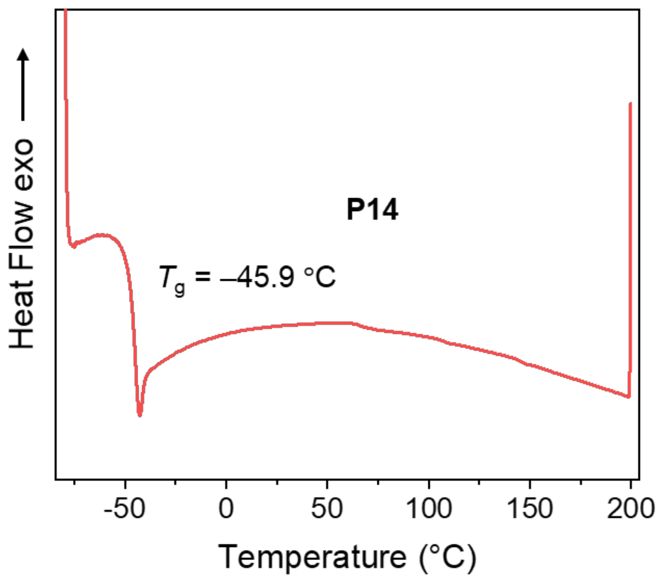

Supplementary Fig. 26 DSC thermogram of **P14** with T_g = –45.9 °C.

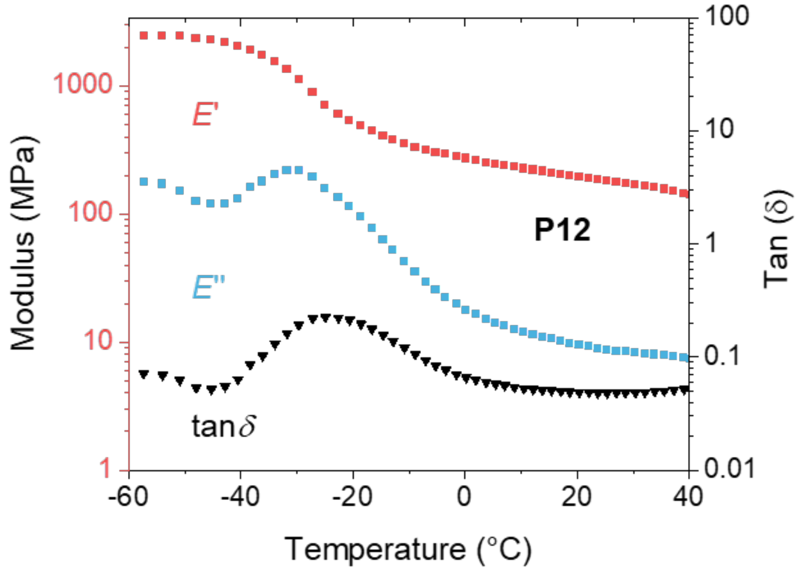

Supplementary Fig. 27 Overlay of storage modulus *E*′, loss modulus *E*″, and tan*δ* for **P12** characterized by DMA.

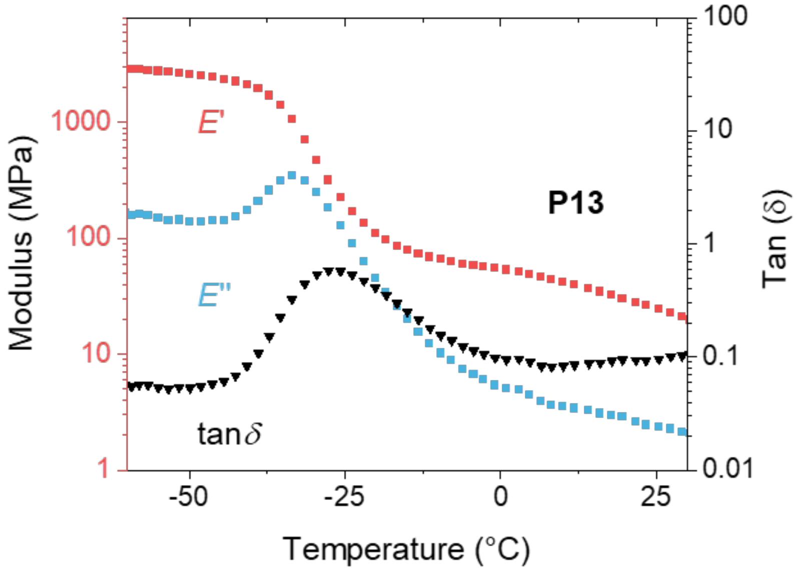

Supplementary Fig. 28 Overlay of storage modulus E', loss modulus E'', and tanδ for **P13** characterized by DMA.

9. ^{1}H NMR spectra of polytrithiocarbonates containing disulfide bonds

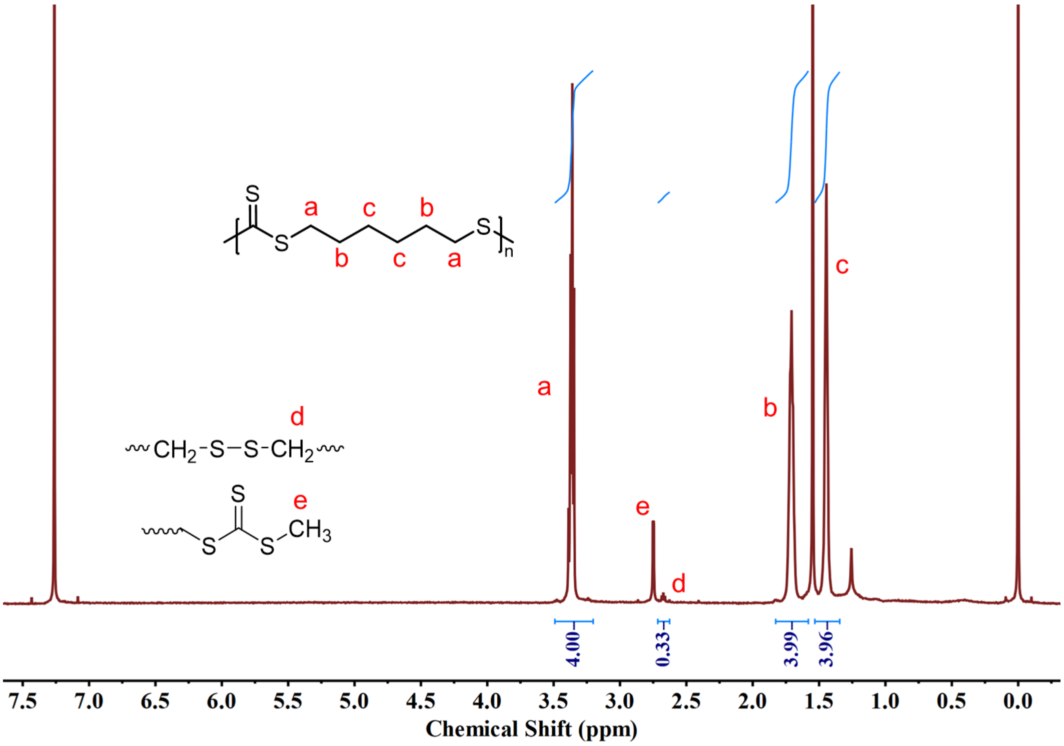

Supplementary Fig. 29 ^{1}H NMR spectrum of **P2** containing disulfide bonds at 40 °C in $CDCl_3$.

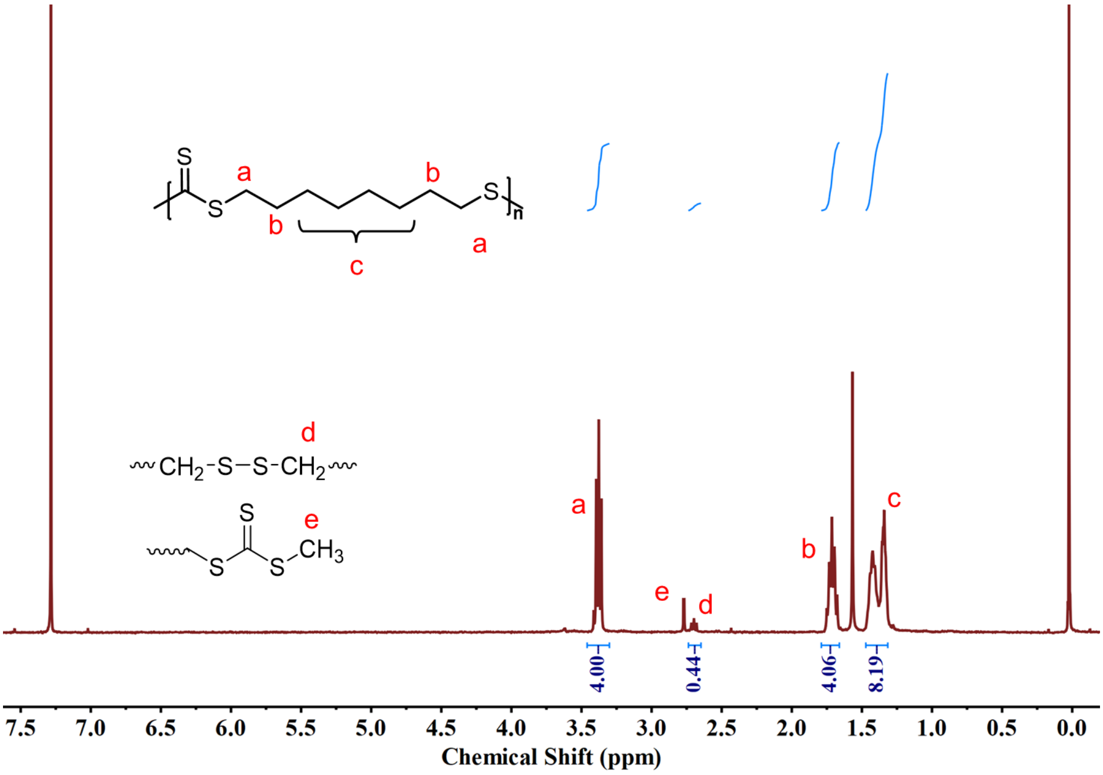

Supplementary Fig. 30 ^{1}H NMR spectrum of **P3** containing disulfide bonds at 40 °C in $CDCl_3$.

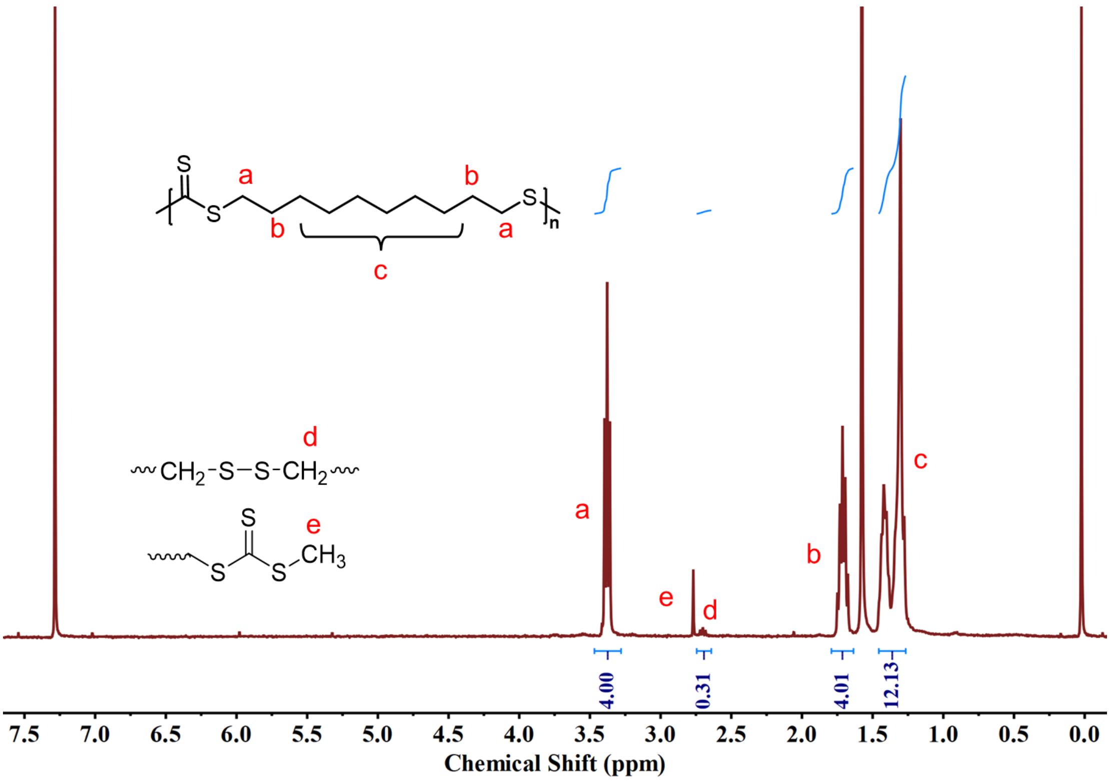

Supplementary Fig. 31 ^{1}H NMR spectrum of **P4** containing disulfide bonds at 40 °C in $CDCl_3$.

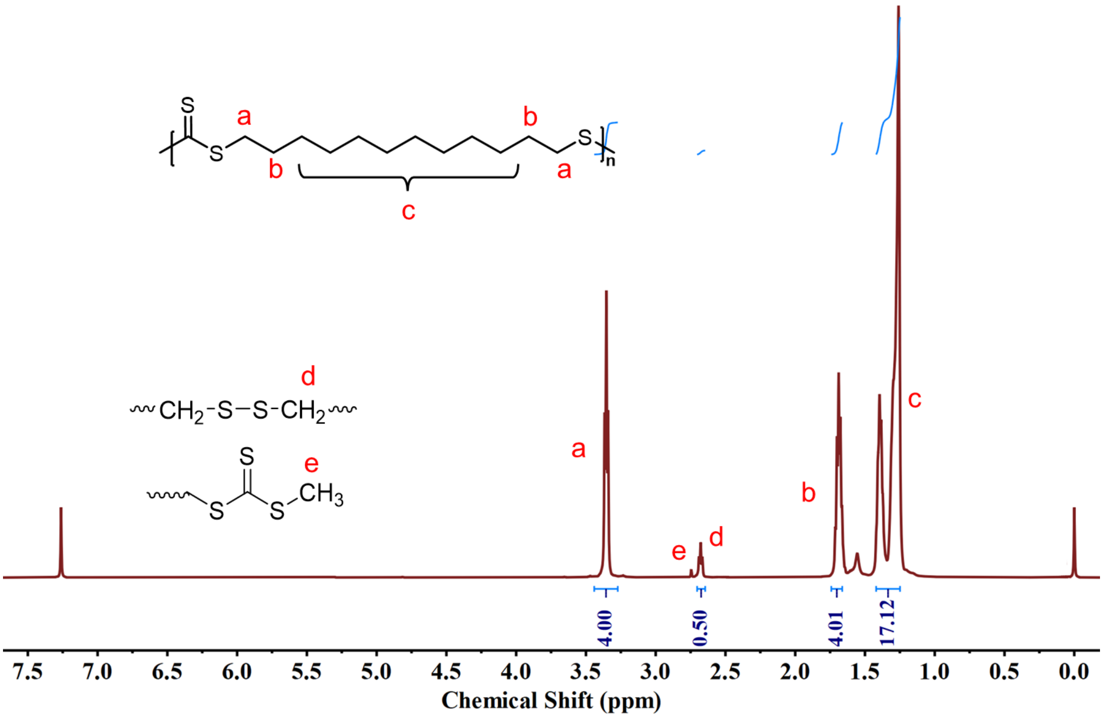

Supplementary Fig. 32 ^{1}H NMR spectrum of **P5** containing disulfide bonds at 40 °C in $CDCl_3$.

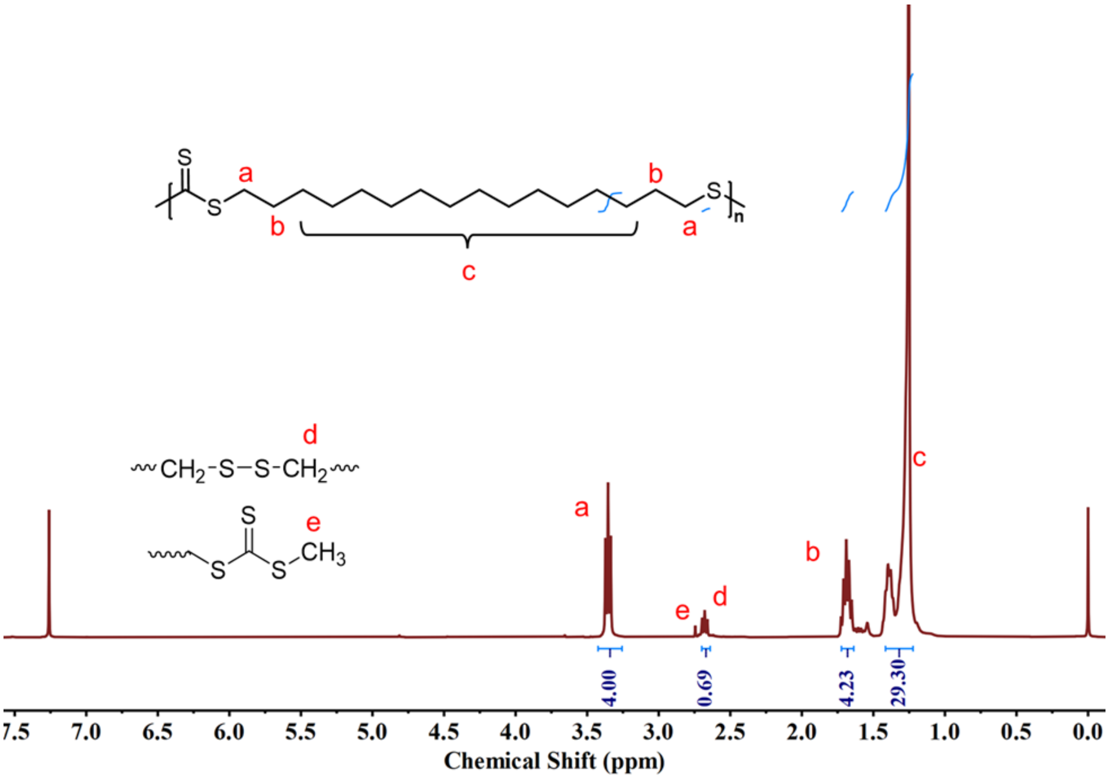

Supplementary Fig. 33 ^{1}H NMR spectrum of **P6** containing disulfide bonds at 40 °C in $CDCl_3$.

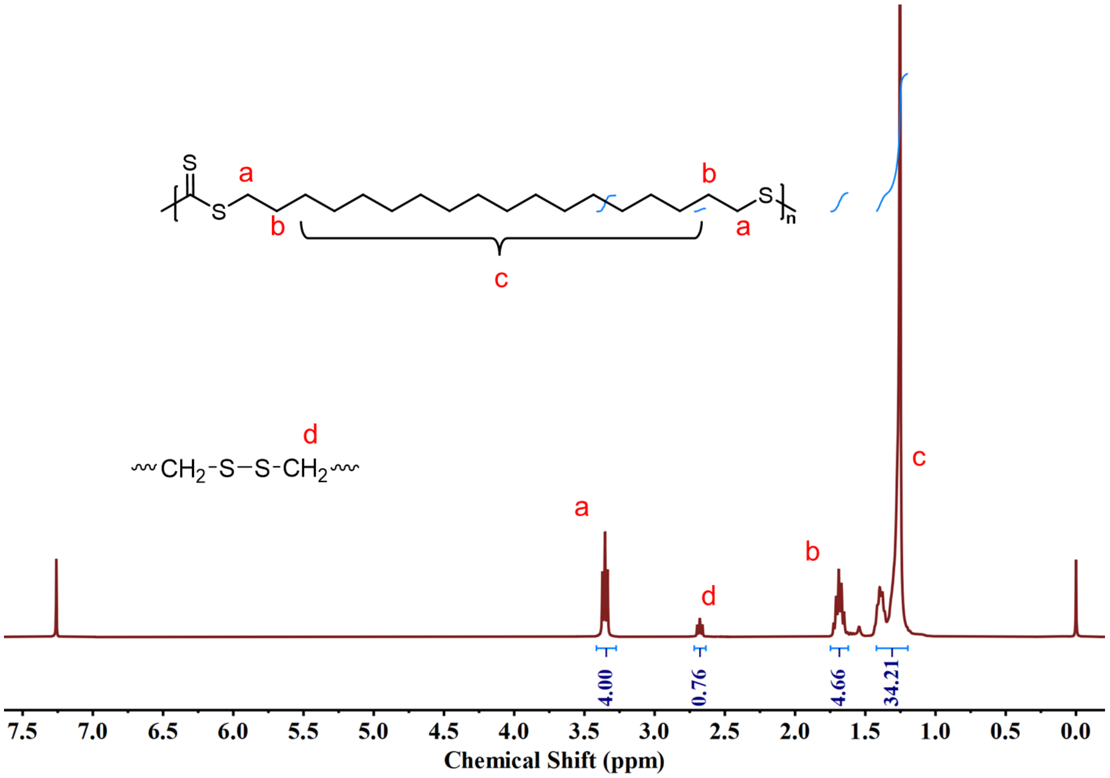

Supplementary Fig. 34 ^{1}H NMR spectrum of **P7** containing disulfide bonds at 40 °C in $CDCl_3$.

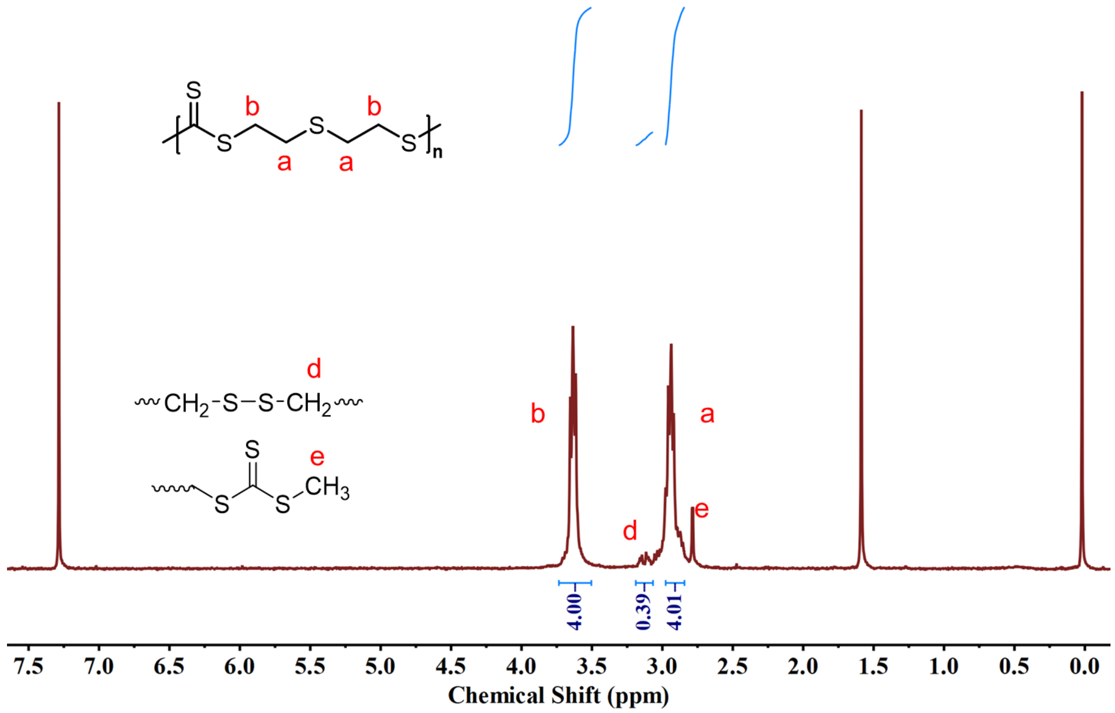

Supplementary Fig. 35 ^{1}H NMR spectrum of **P8** containing disulfide bonds at 40 °C in $CDCl_3$.

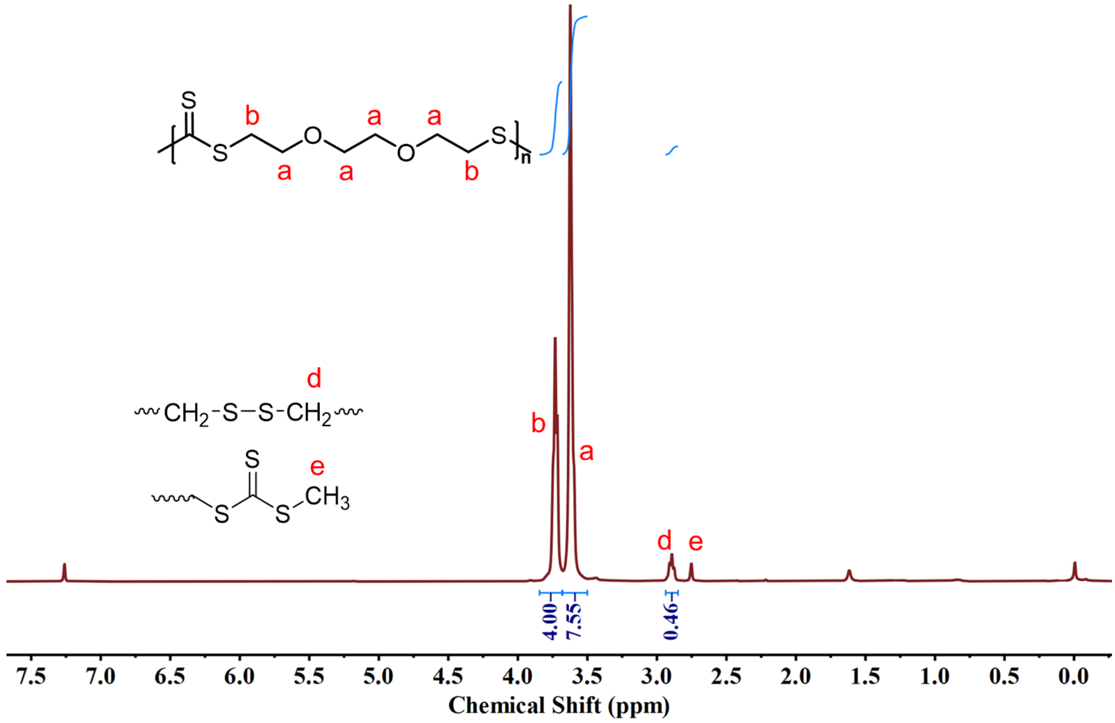

Supplementary Fig. 36 ^{1}H NMR spectrum of **P9** containing disulfide bonds at 40 °C in $CDCl_3$.

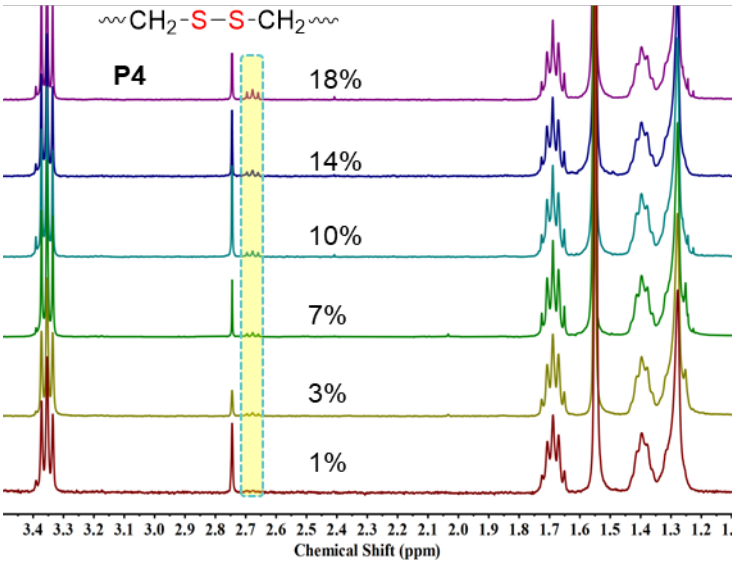

Supplementary Fig. 37 Overlay of ^{1}H NMR spectra (25 °C, $CDCl_3$) of **P4** with different disulfide linkage content ranging from 1% to 18%.

10. Synthesis of vitrimer **P15**

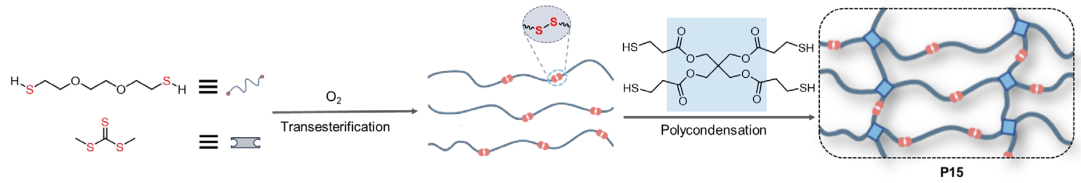

9 (20.0 mmol, 3.64 g, 1.0 equiv.) and DMTC (20.4 mmol, 2.82 g, 1.02 equiv.) were added to the pre-built polycondensation device, followed by charging O_2 (1.0 L) into reaction system through bubbling for 5 min. Then, KH (0.5% equiv.) was added and the flask was immersed in a hot-oil bath (120 °C), the reaction was conducted for 2.0 h with the pressure gradually reduced to70000 Pa. Then, pentaerythritol *tetrakis* (3-mercapto-propionate) (0.2 mmol, 97.3 mg, 0.01 equiv.) was added to the reaction system. The polycondensation was continued as the representative polycondensation procedures described above.

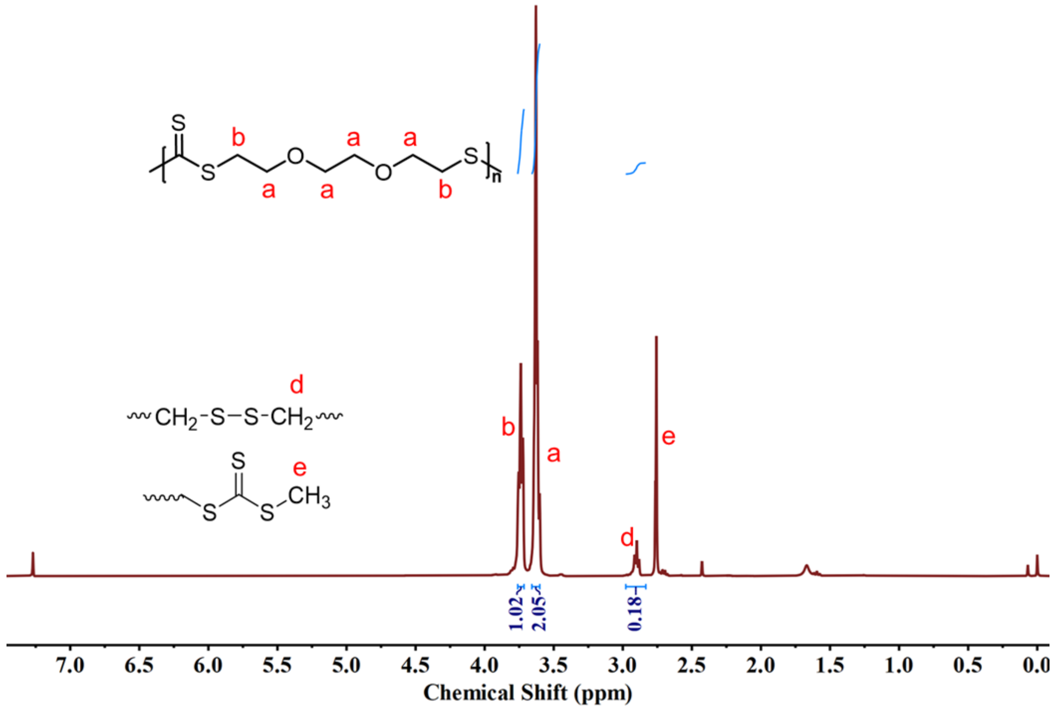

Supplementary Fig. 38 ^{1}H NMR spectrum of **P9** oligomers with 18% disulfide linkage content at 25 °C in $CDCl_3$.

11. Closed-loop recycling of polytrithiocarbonates

General procedure for the depolymerization of polytrithiocarbonates

Taking **P6** as an example, the recycling of **P6** (4.5 g) was performed in a 35 mL pressure-resistant bottle in the presence of KOH (230 mg, 10 wt%) and CH_3SH (14 g, 300 wt%) under nitrogen atmosphere at 50 °C for 24 h. Upon cooling, the mixture of DMTC and 1,16-hexadecanedithiol were obtained after washing by water and rotary evaporating. Pure dithiols and DMTC could be recovered with a yield of no less than 97% after recrystallization. **P6** can be synthesize from the recovered two monomers or the directly mixture without separation.

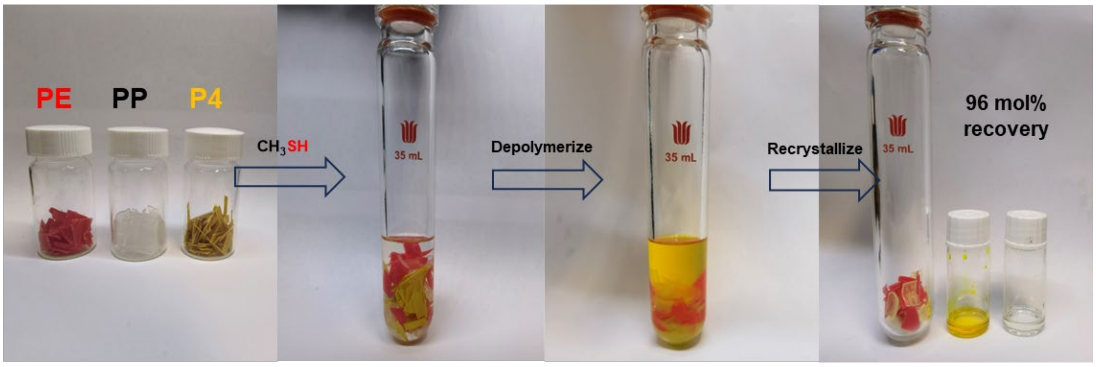

Supplementary Fig. 39 Use of a mixture of PP (polypropylene), HDPE and **P4** as an example of a potential polyolefin waste stream. After being treated with potassium hydroxide in MeSH at 50 °C for 24 h, DMTC and dithiols were recovered from mixed polyolefin plastics waste, yields 96%.

12. NMR of DMTC, dithiols and polytrithiocarbonates

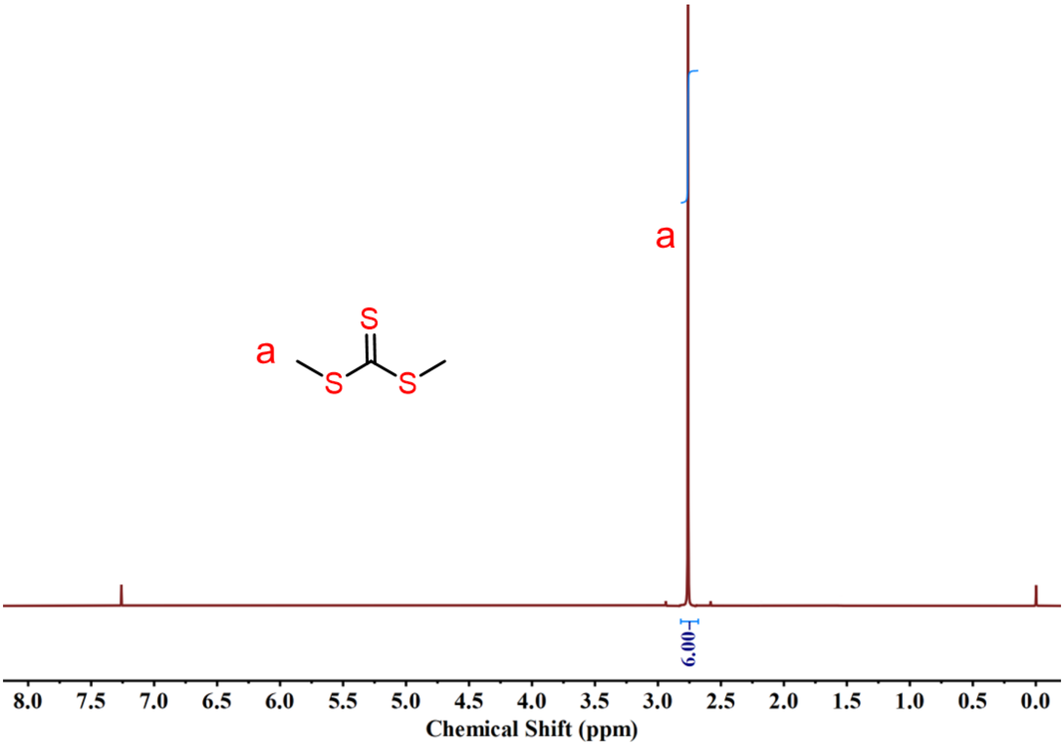

Supplementary Fig. 40 ^{1}H NMR spectrum of DMTC at 25 °C in $CDCl_3$.

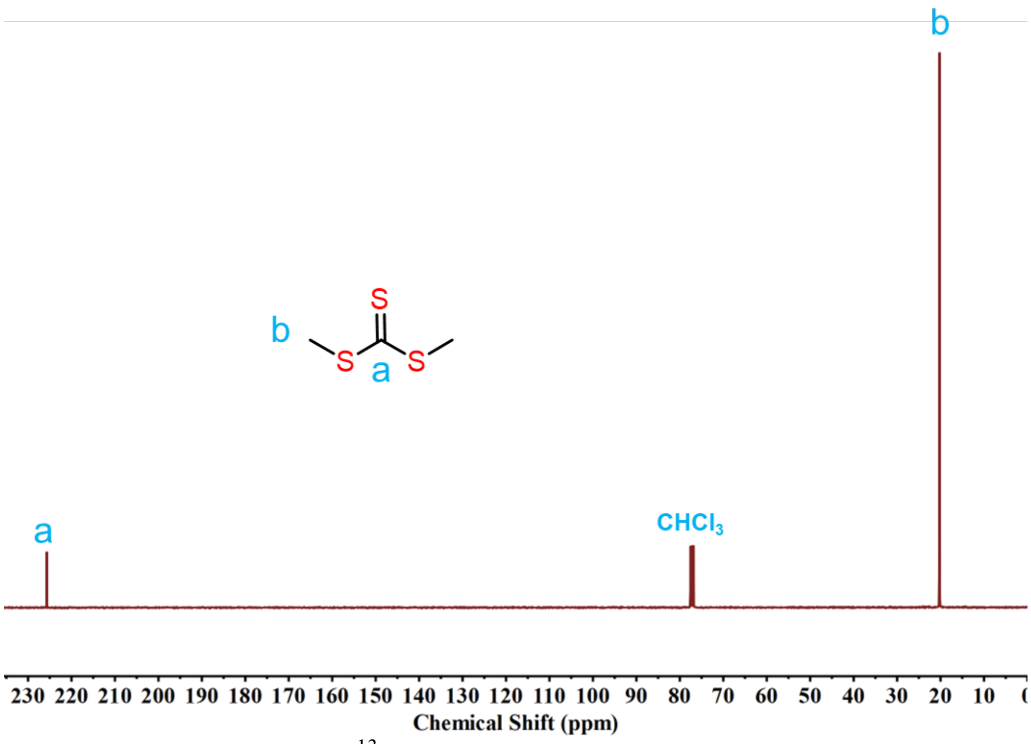

Supplementary Fig. 41 ^{13}C NMR spectrum of DMTC at 25 °C in $CDCl_3$.

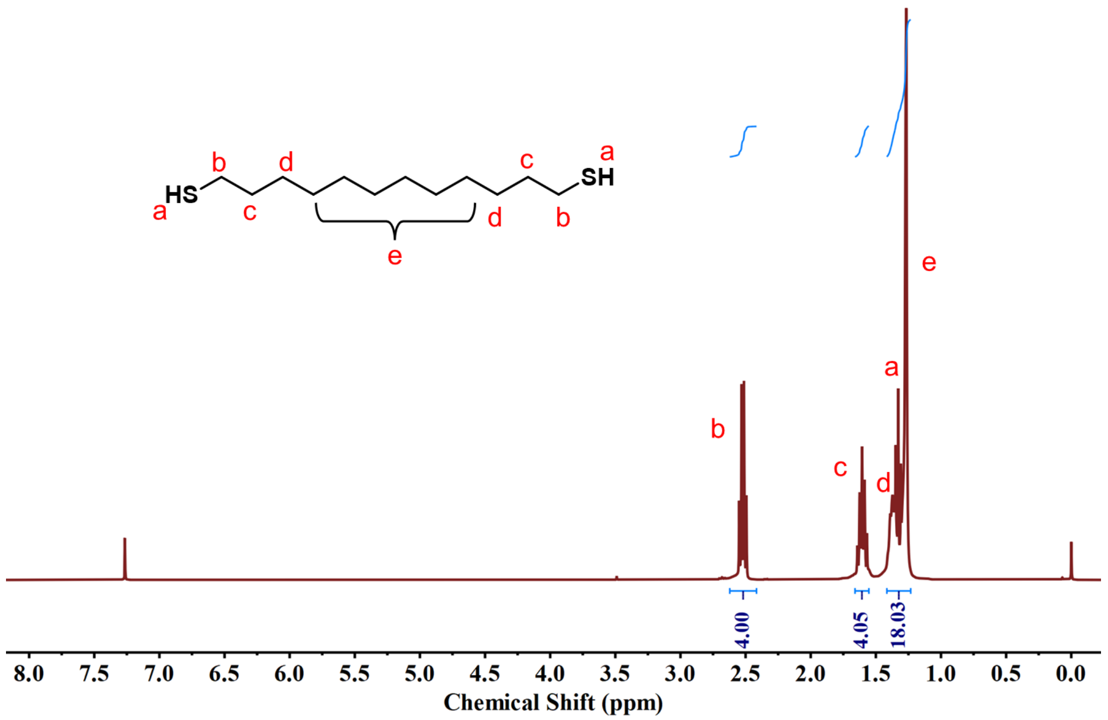

Supplementary Fig. 42 ^{1}H NMR spectrum of 1,12-dodecanedithiol at 25 °C in $CDCl_3$.

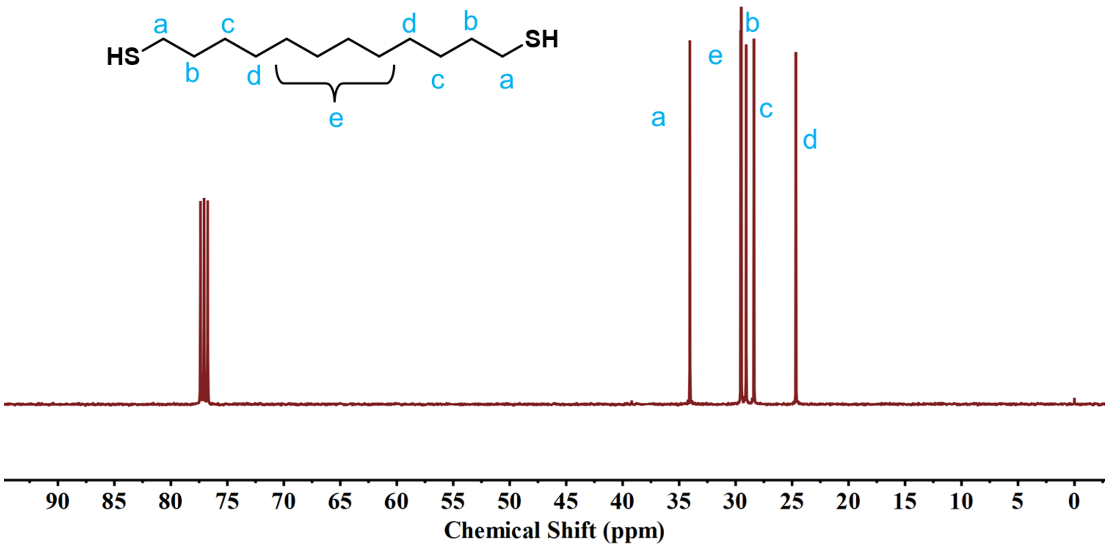

Supplementary Fig. 43 ^{13}C NMR spectrum of 1,12-dodecanedithiol at 25 °C in $CDCl_3$.

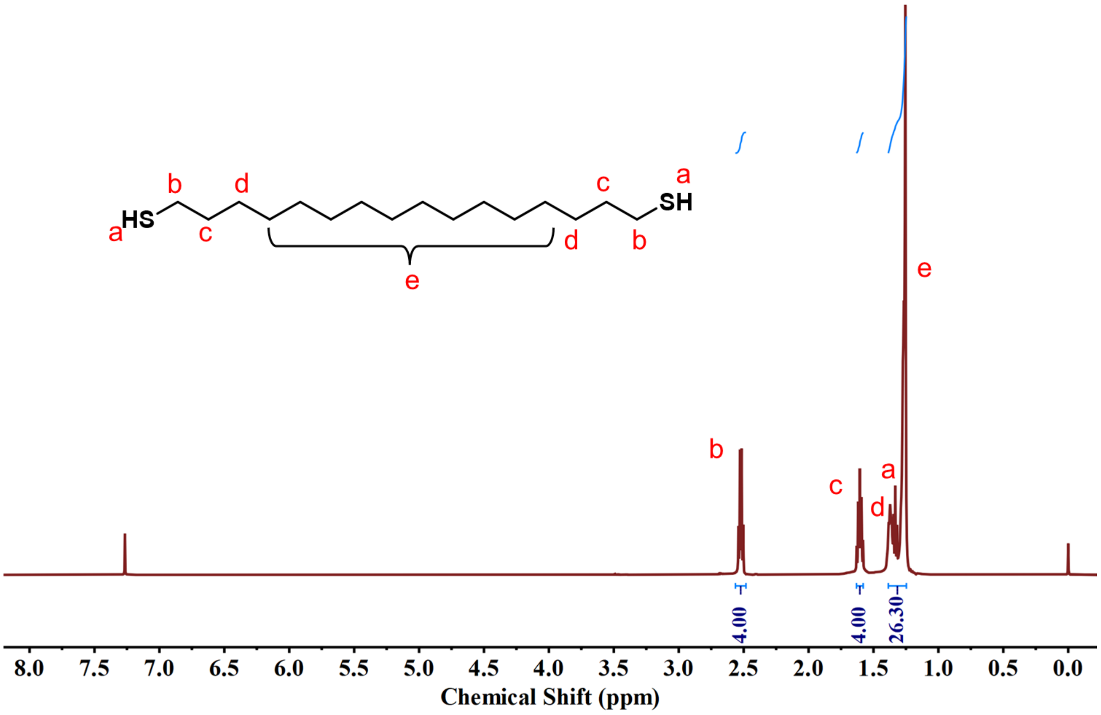

Supplementary Fig. 44 ^{1}H NMR spectrum of 1,16-hexadecanedithiol at 25 °C in $CDCl_3$.

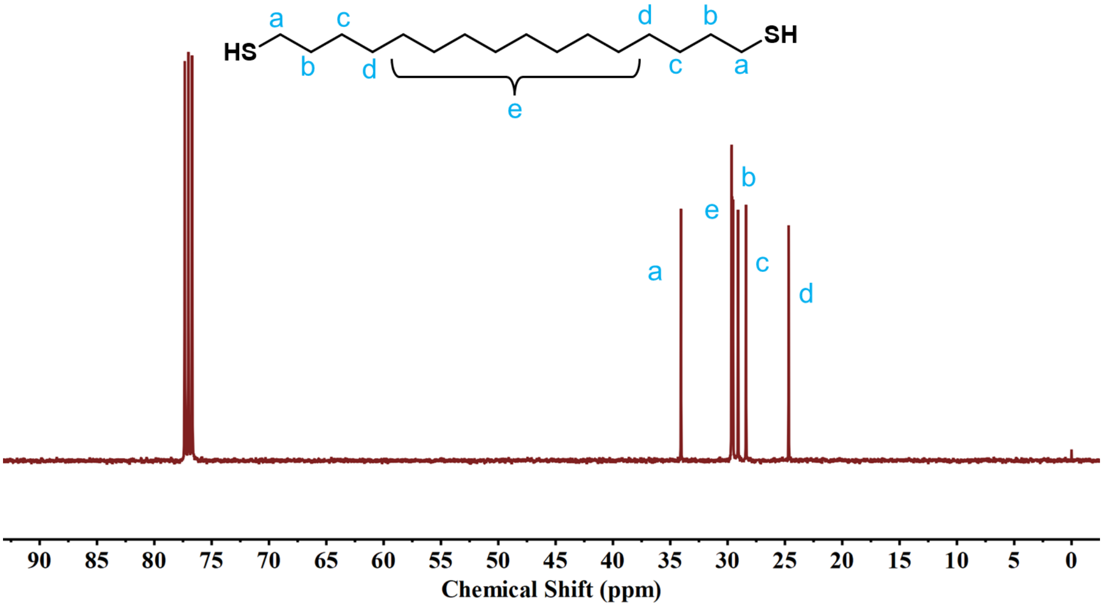

Supplementary Fig. 45 ^{13}C NMR spectrum of 1,16-hexadecanedithiol at 25 °C in $CDCl_3$.

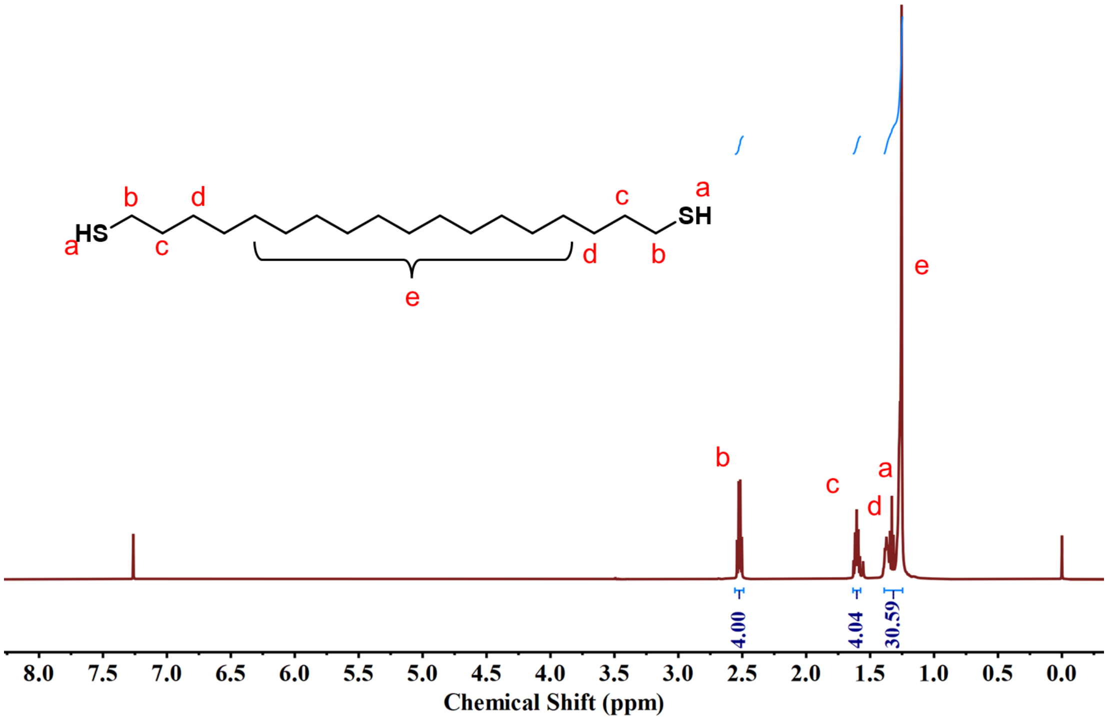

Supplementary Fig. 46 ^{1}H NMR spectrum of 1,18-octadecanedithiol at 25 °C in $CDCl_3$.

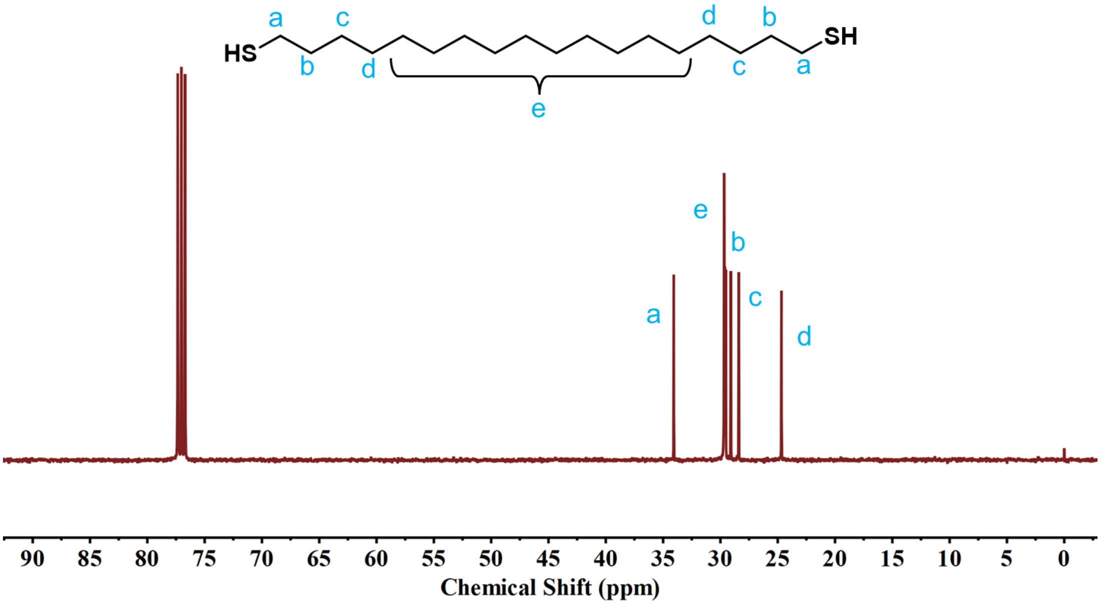

Supplementary Fig. 47 ^{13}C NMR spectrum of 1,18-octadecanedithiol at 25 °C in $CDCl_3$.

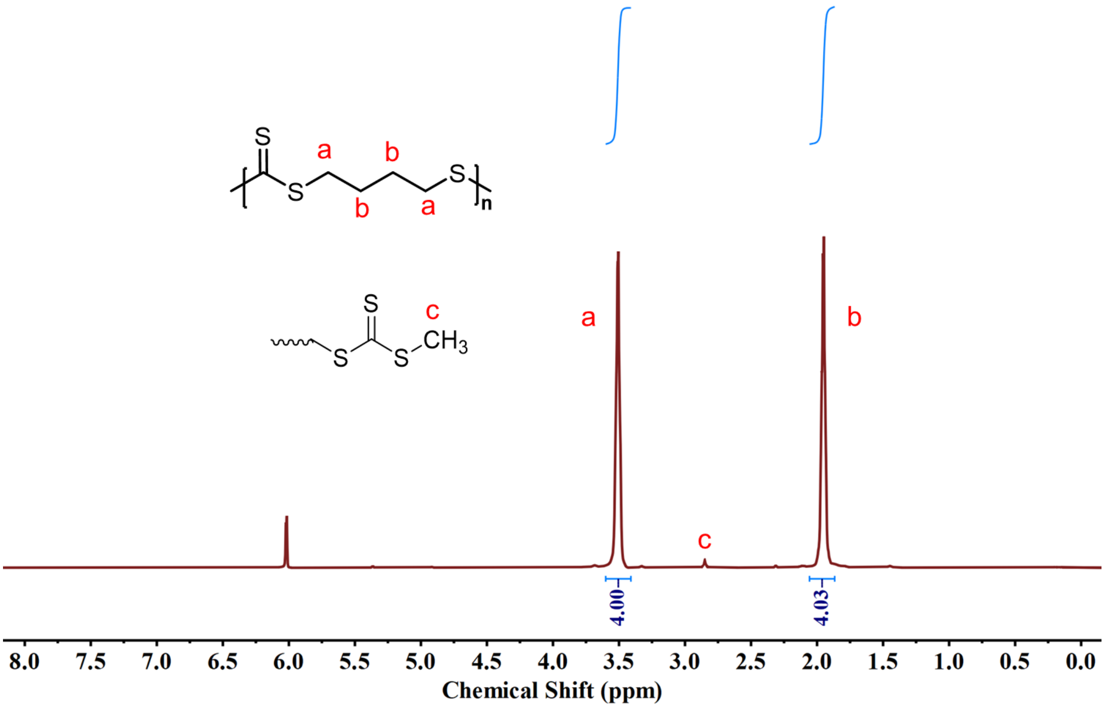

Supplementary Fig. 48 ^{1}H NMR spectrum of **P1** at 120 °C in tetrachloroethane-d_2.

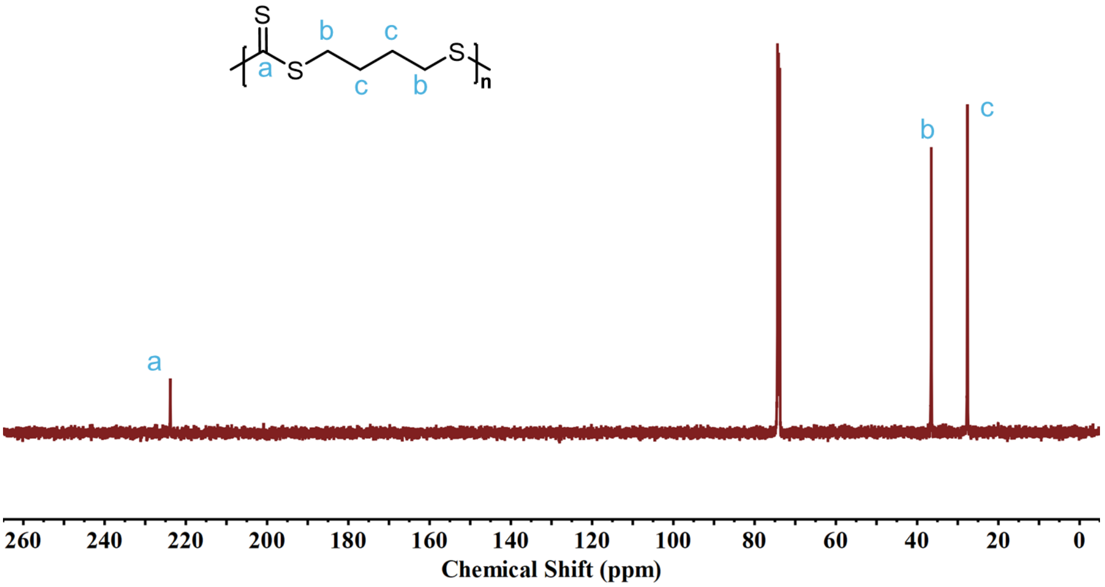

Supplementary Fig. 49 ^{13}C NMR spectrum of **P1** at 120 °C in tetrachloroethane-d_2.

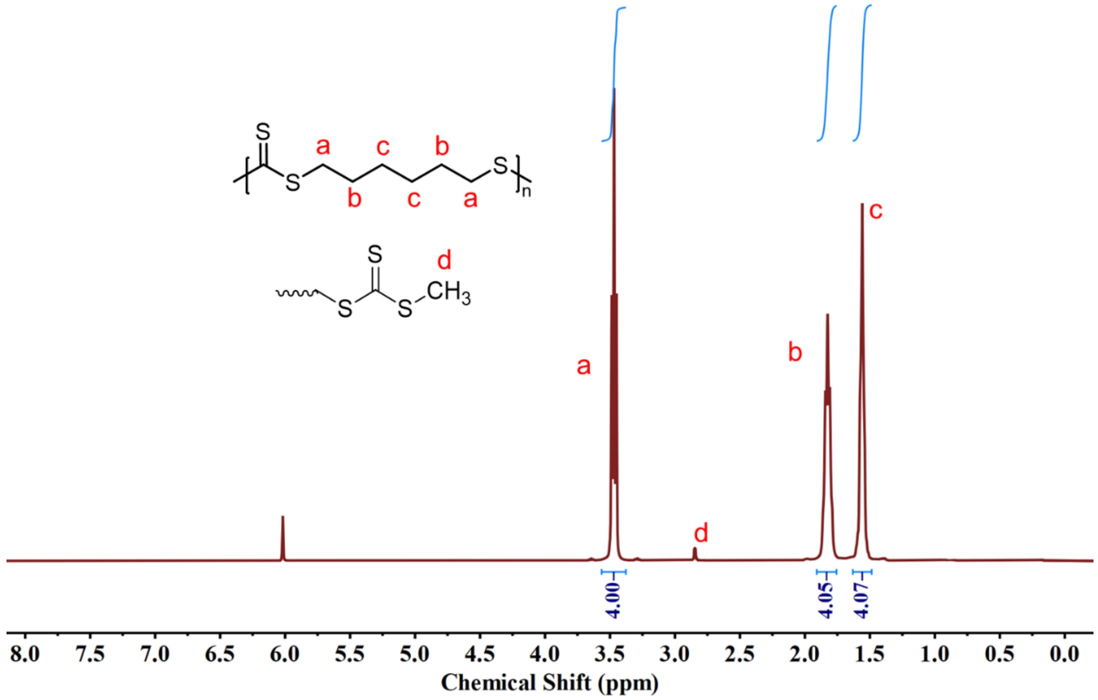

Supplementary Fig. 50 ^{1}H NMR spectrum of **P2** at 120 °C in tetrachloroethane-d_2.

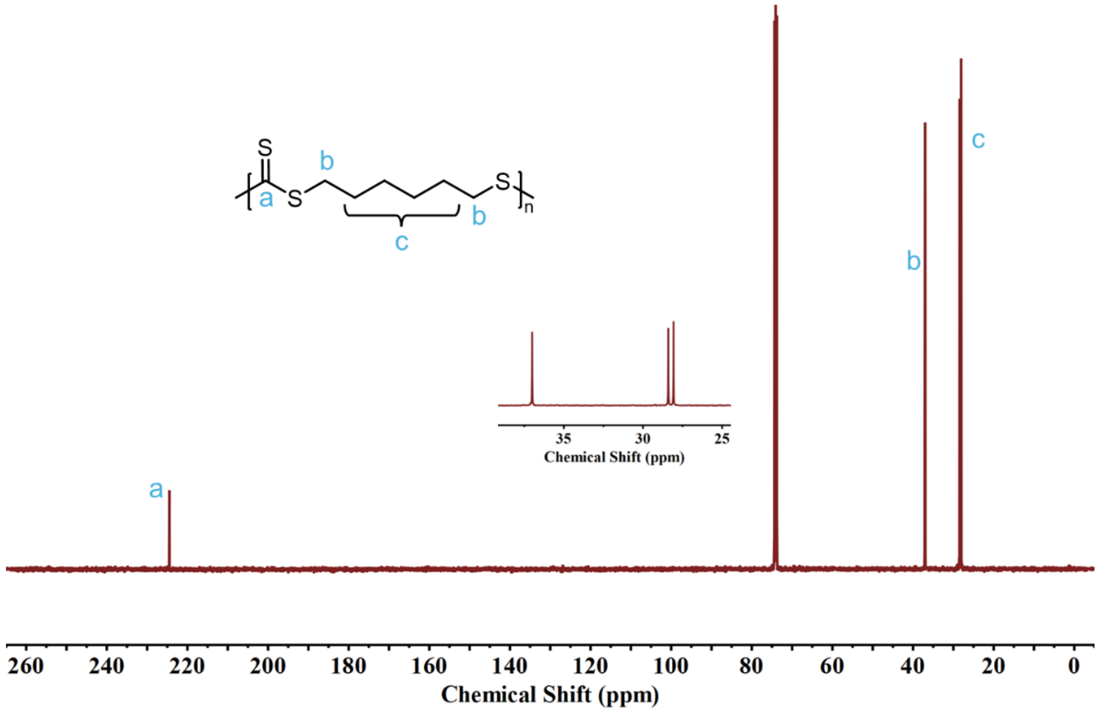

Supplementary Fig. 51 ^{13}C NMR spectrum of **P2** at 120 °C in tetrachloroethane-d_2.

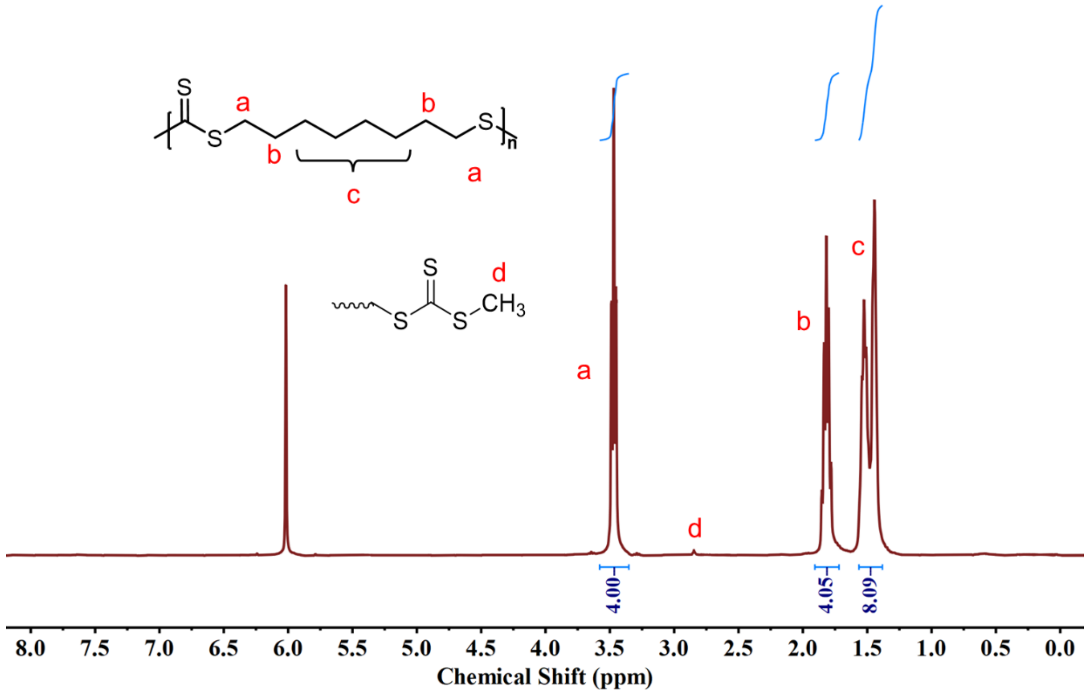

Supplementary Fig. 52 ^{1}H NMR spectrum of **P3** at 120 °C in tetrachloroethane-d_2.

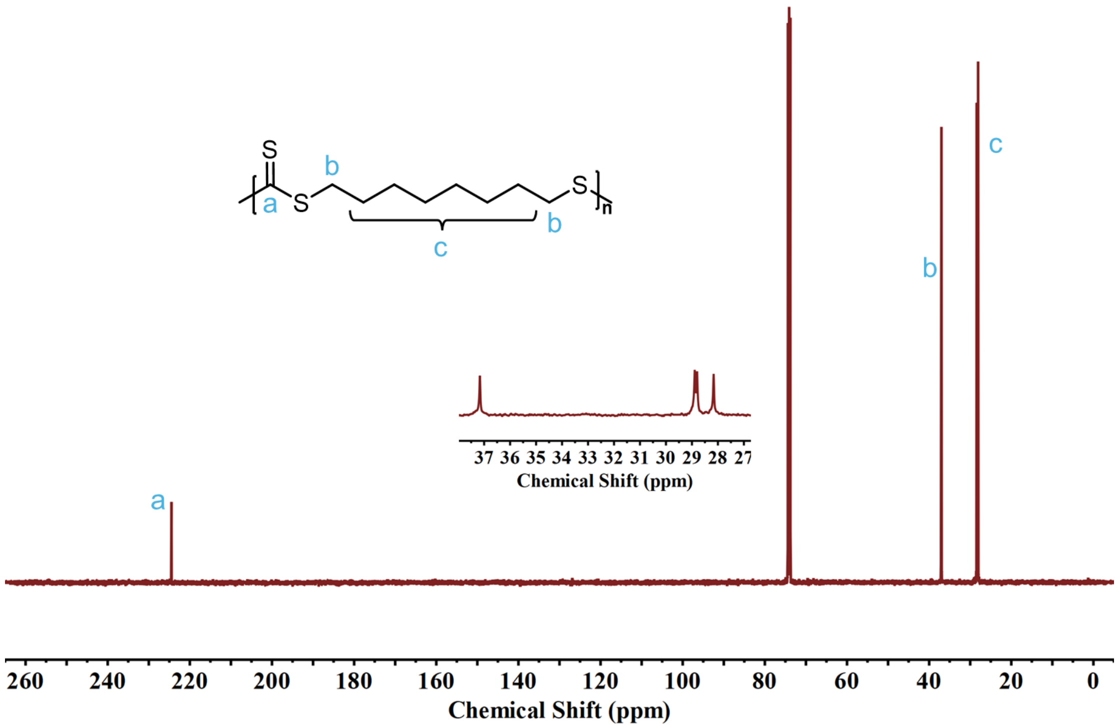

Supplementary Fig. 53 ^{13}C NMR spectrum of **P3** at 120 °C in tetrachloroethane-d_2.

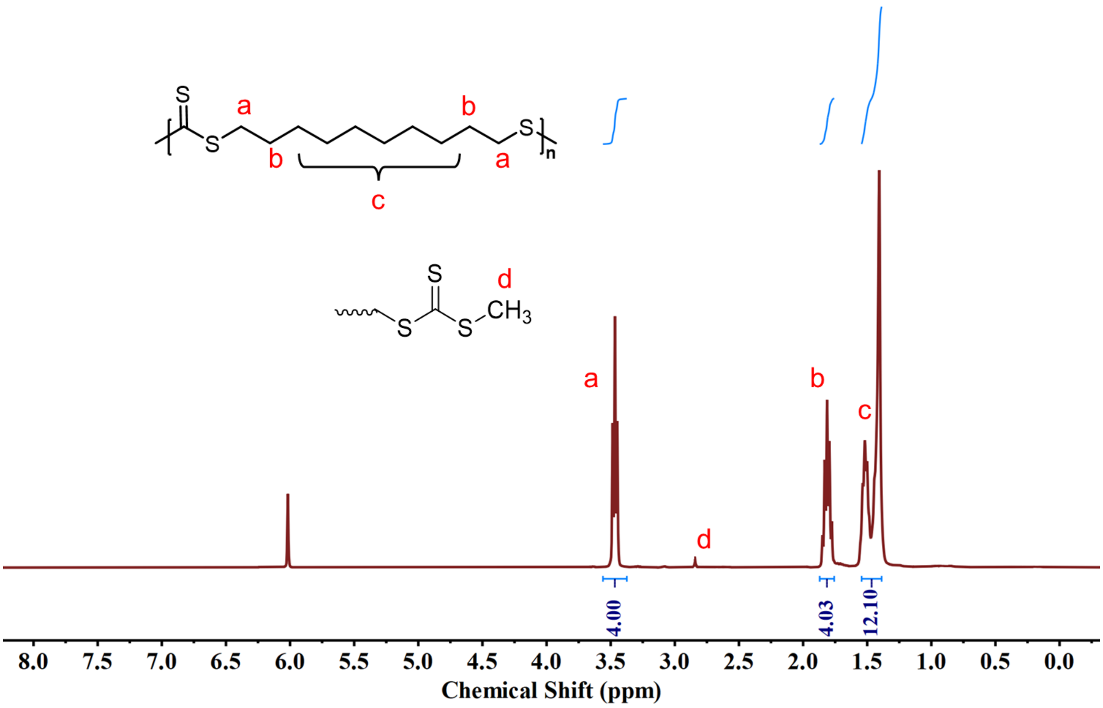

Supplementary Fig. 54 ^{1}H NMR spectrum of **P4** at 120 °C in tetrachloroethane-d_2.

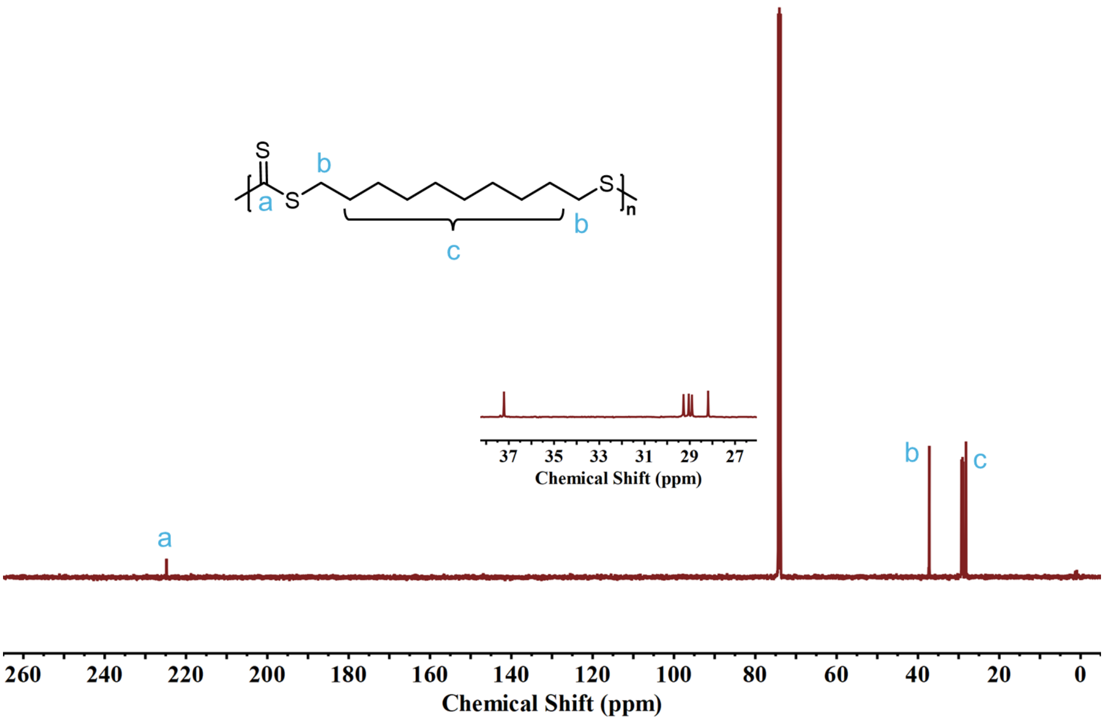

Supplementary Fig. 55 ^{13}C NMR spectrum of **P4** at 120 °C in tetrachloroethane-d_2.

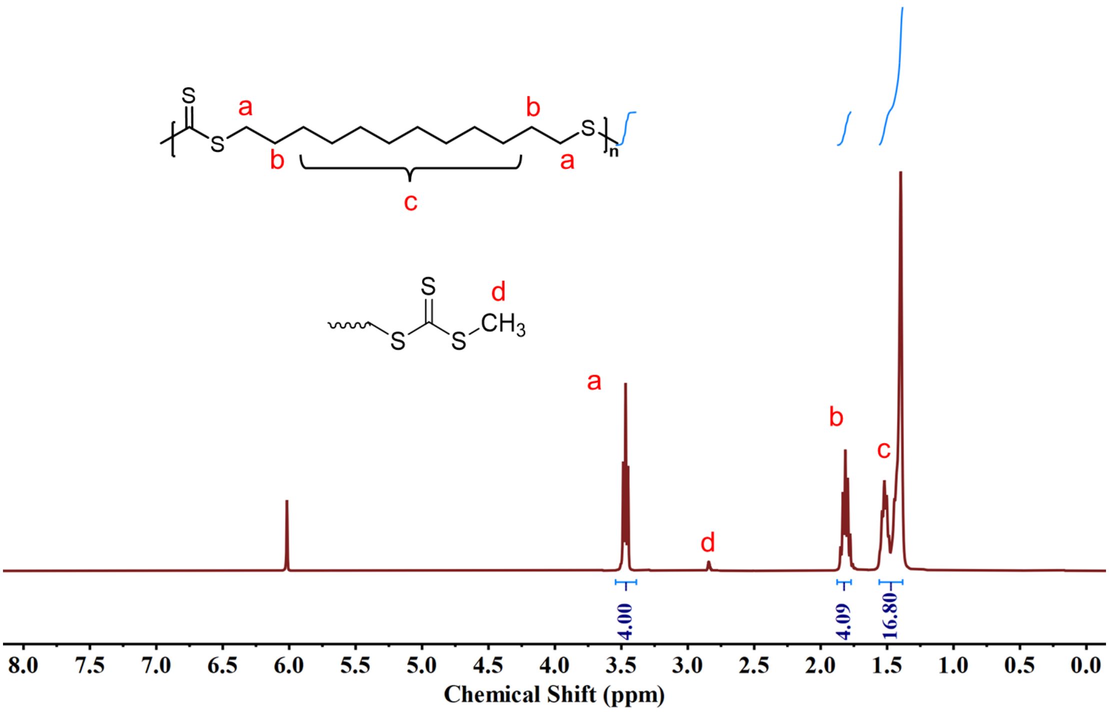

Supplementary Fig. 56 ^{1}H NMR spectrum of **P5** at 120 °C in tetrachloroethane-d_2.

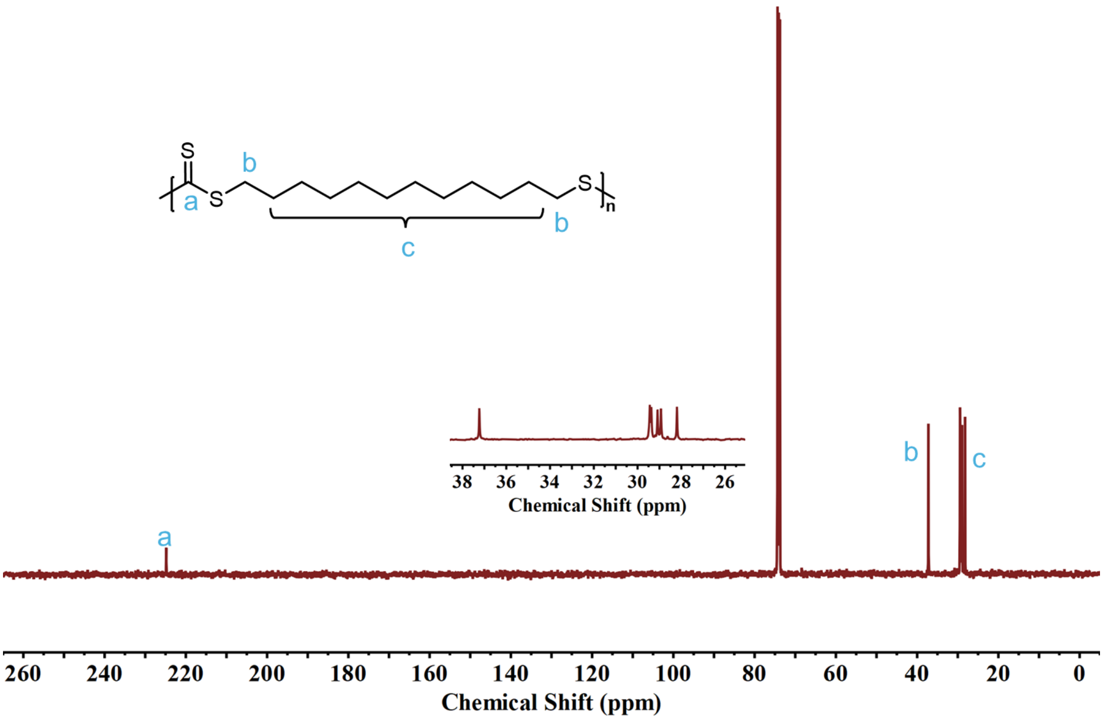

Supplementary Fig. 57 ^{13}C NMR spectrum of **P5** at 120 °C in tetrachloroethane-d_2.

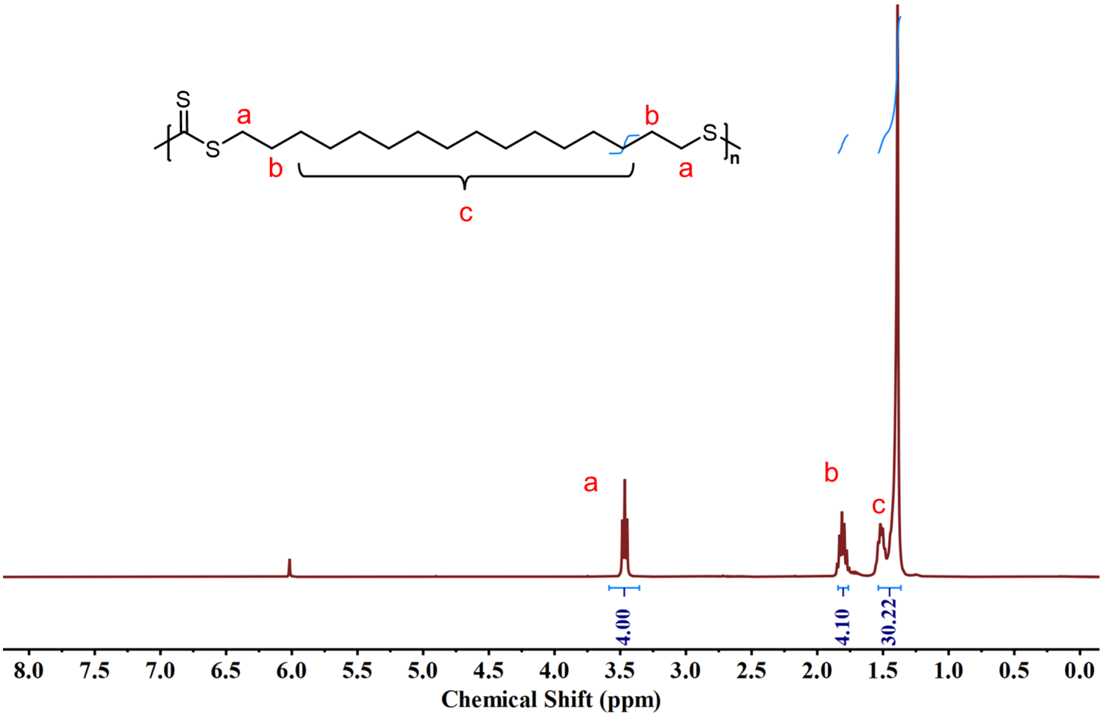

Supplementary Fig. 58 ^{1}H NMR spectrum of **P6** at 120 °C in tetrachloroethane-d_2.

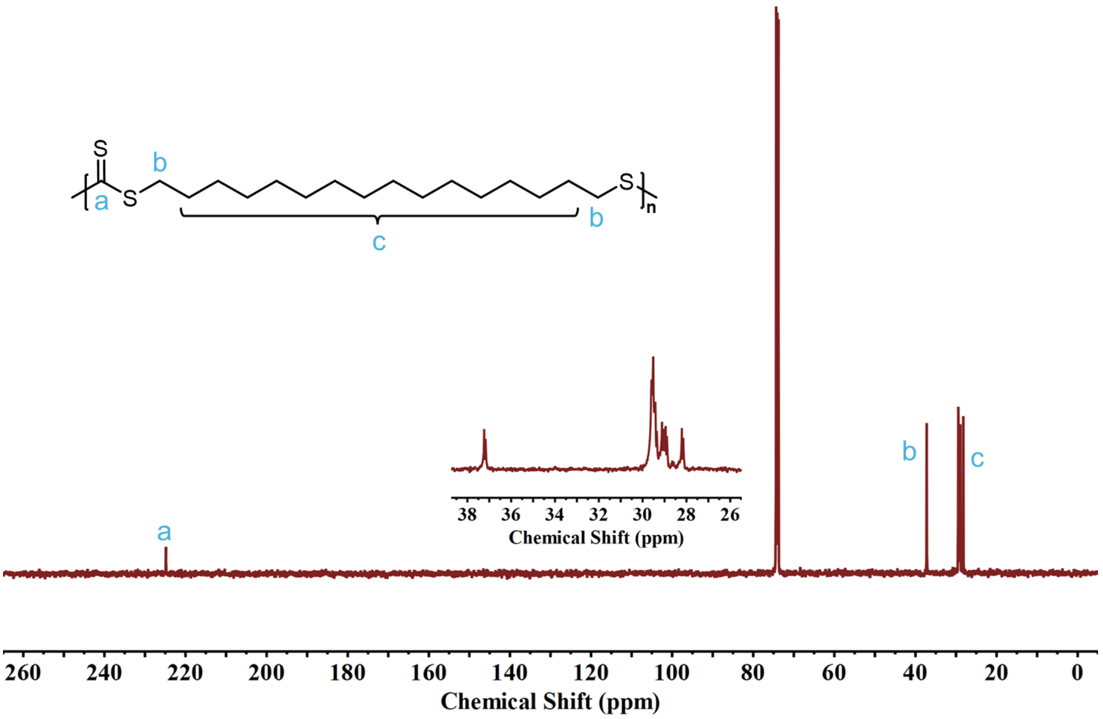

Supplementary Fig. 59 ^{13}C NMR spectrum of **P6** at 120 °C in tetrachloroethane-d_2.

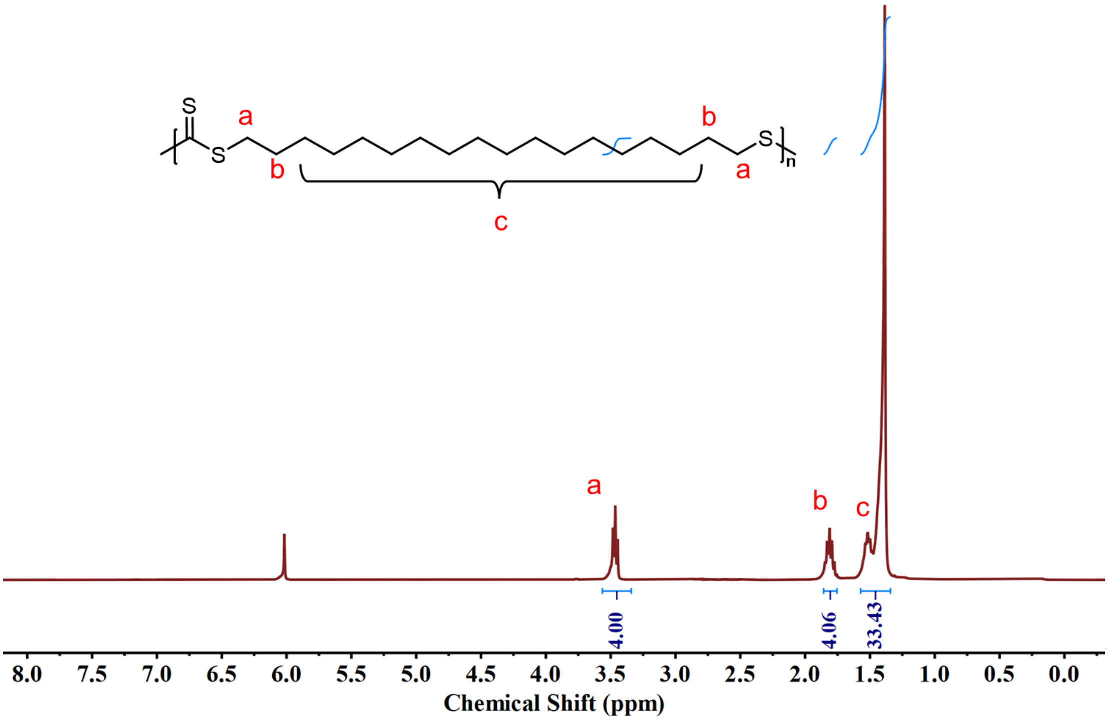

Supplementary Fig. 60 ^{1}H NMR spectrum of **P7** at 120 °C in tetrachloroethane-d_2.

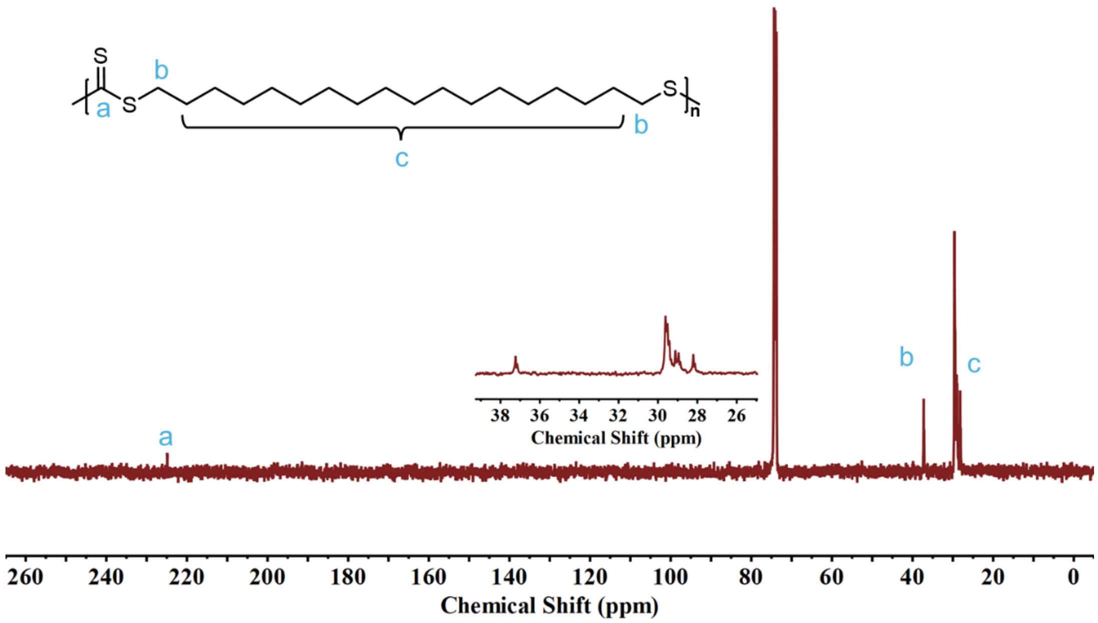

Supplementary Fig. 61 ^{13}C NMR spectrum of **P7** at 120 °C in tetrachloroethane-d_2.

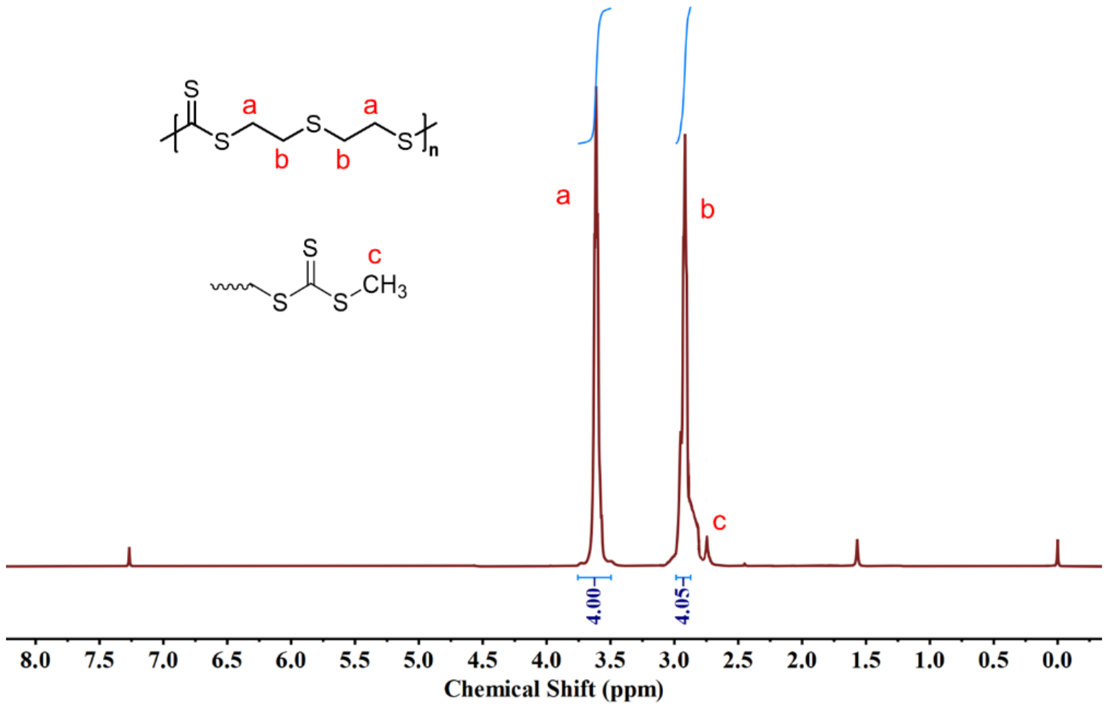

Supplementary Fig. 62 ^{1}H NMR spectrum of **P8** at 40 °C in $CDCl_3$.

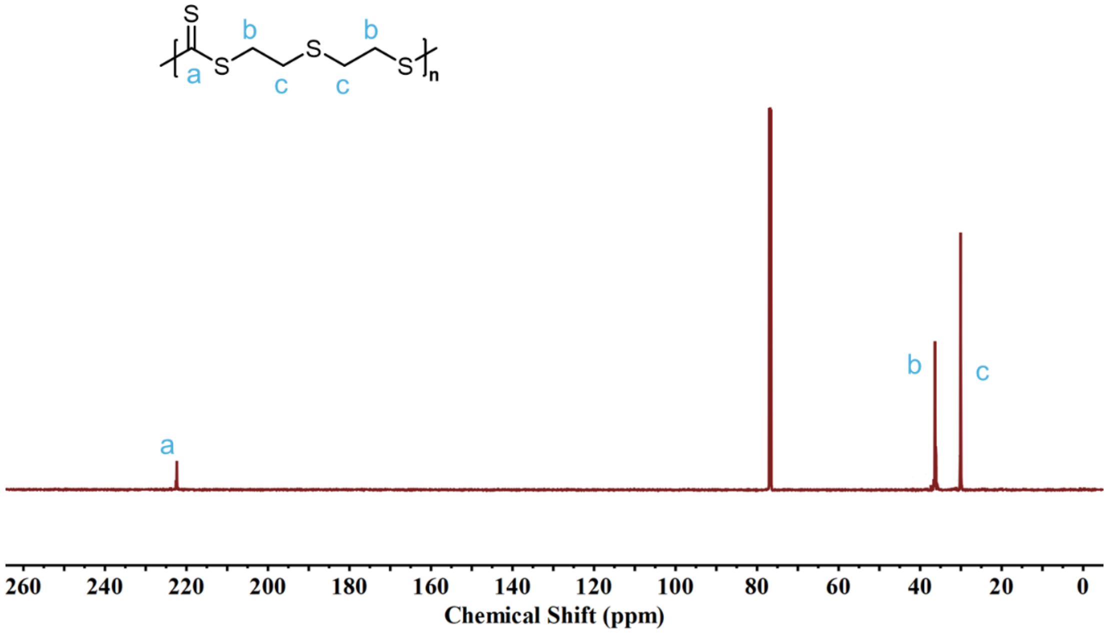

Supplementary Fig. 63 ^{13}C NMR spectrum of **P8** at 40 °C in $CDCl_3$.

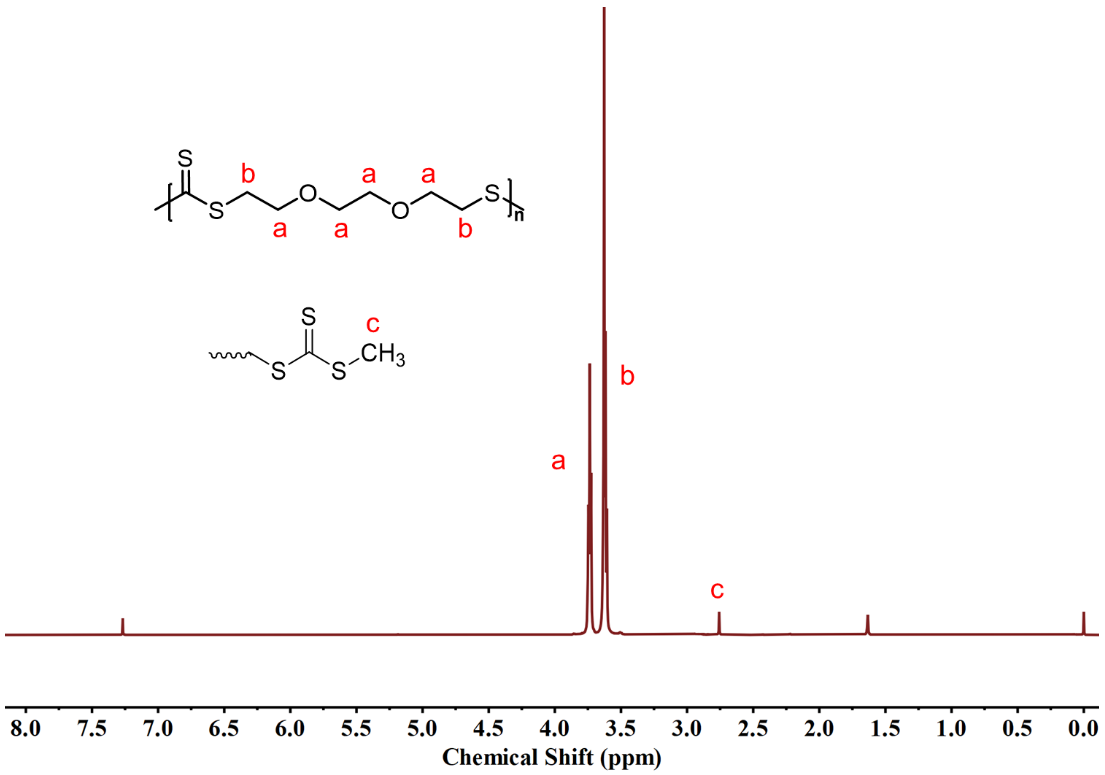

Supplementary Fig. 64 ^{1}H NMR spectrum of **P9** at 25 °C in $CDCl_3$.

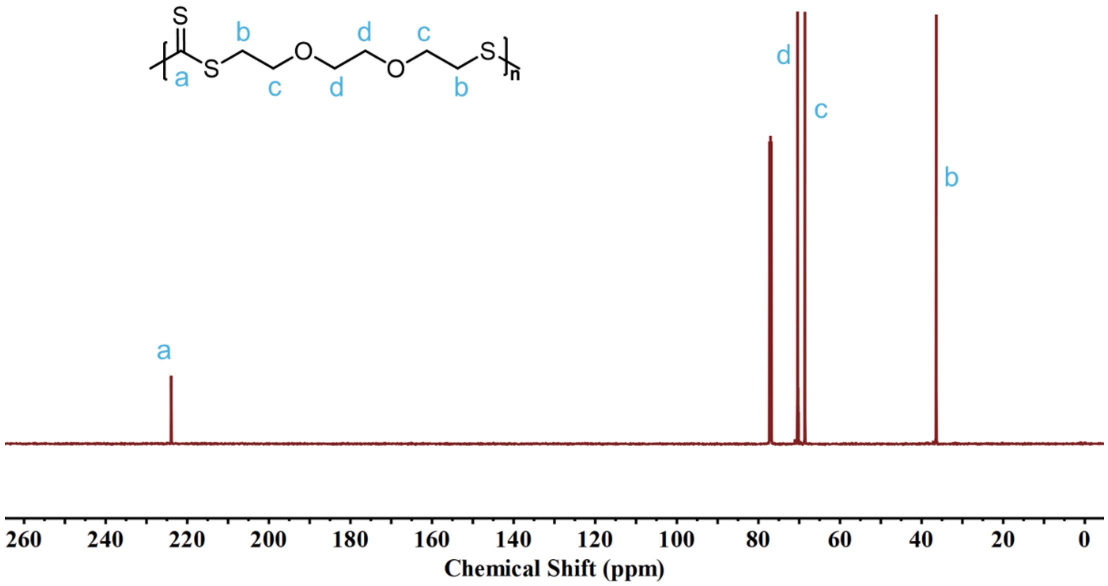

Supplementary Fig. 65 ^{13}C NMR spectrum of **P9** at 25 °C in $CDCl_3$

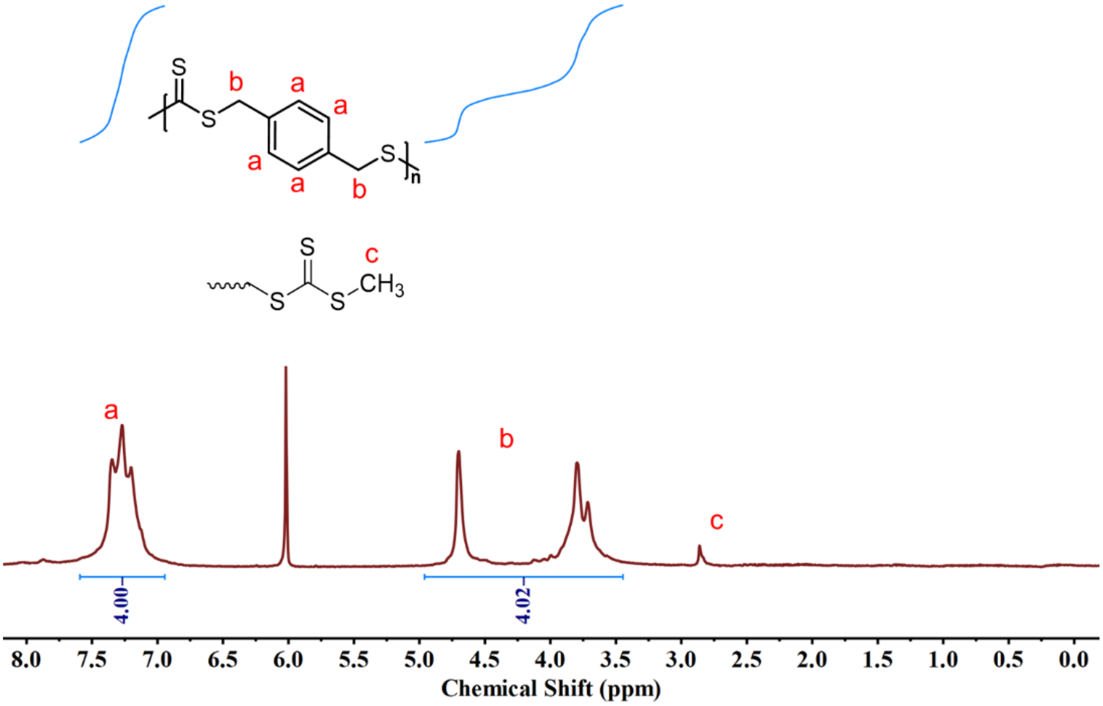

Supplementary Fig. 66 The ^{1}H NMR spectrum of **P10** at 120 °C in tetrachloroethane-d_2.

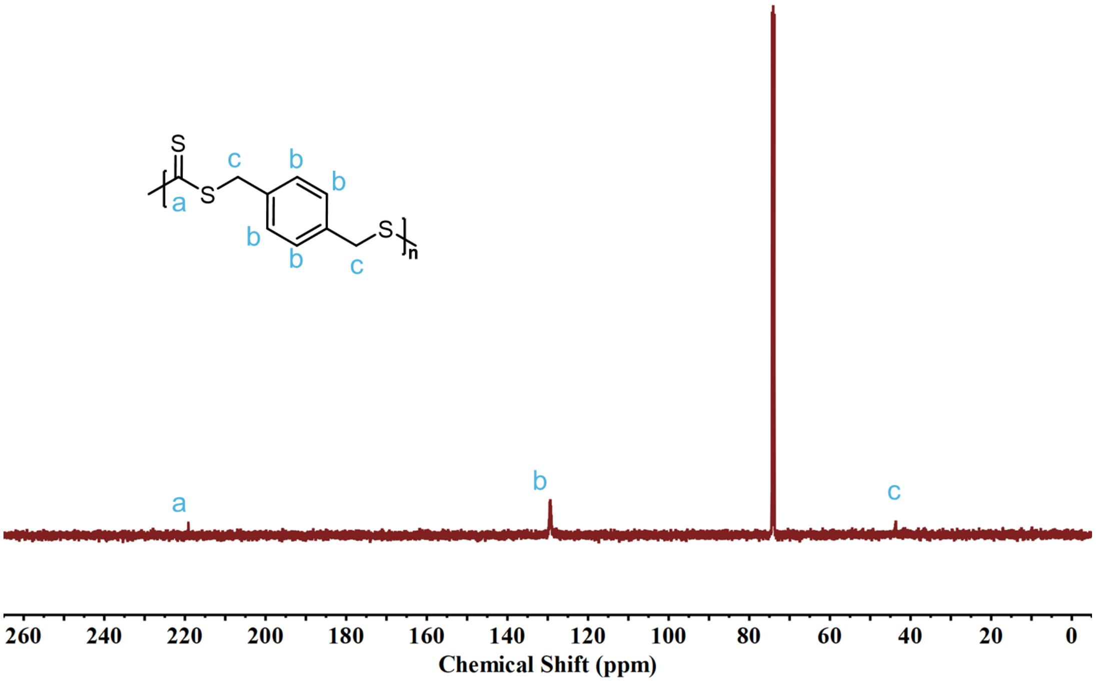

Supplementary Fig. 67 The ^{13}C NMR spectrum of **P10** at 120 °C in tetrachloroethane-d_2.

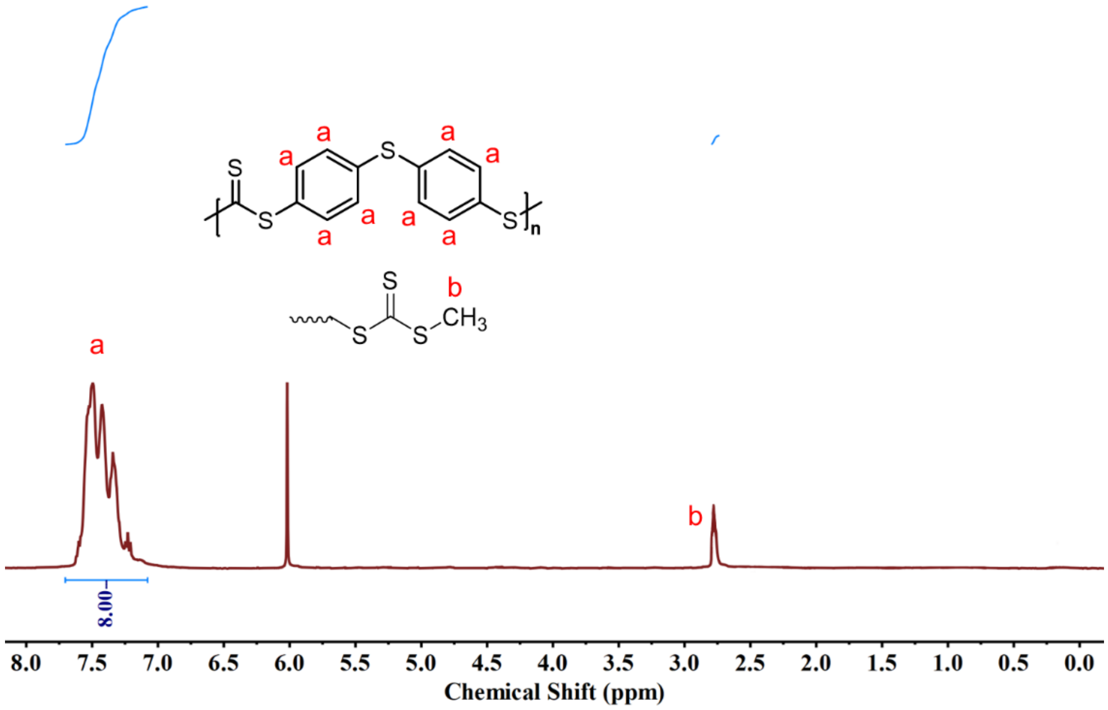

Supplementary Fig. 68 The ^{1}H NMR spectrum of **P11** at 120 °C in tetrachloroethane-d_2.

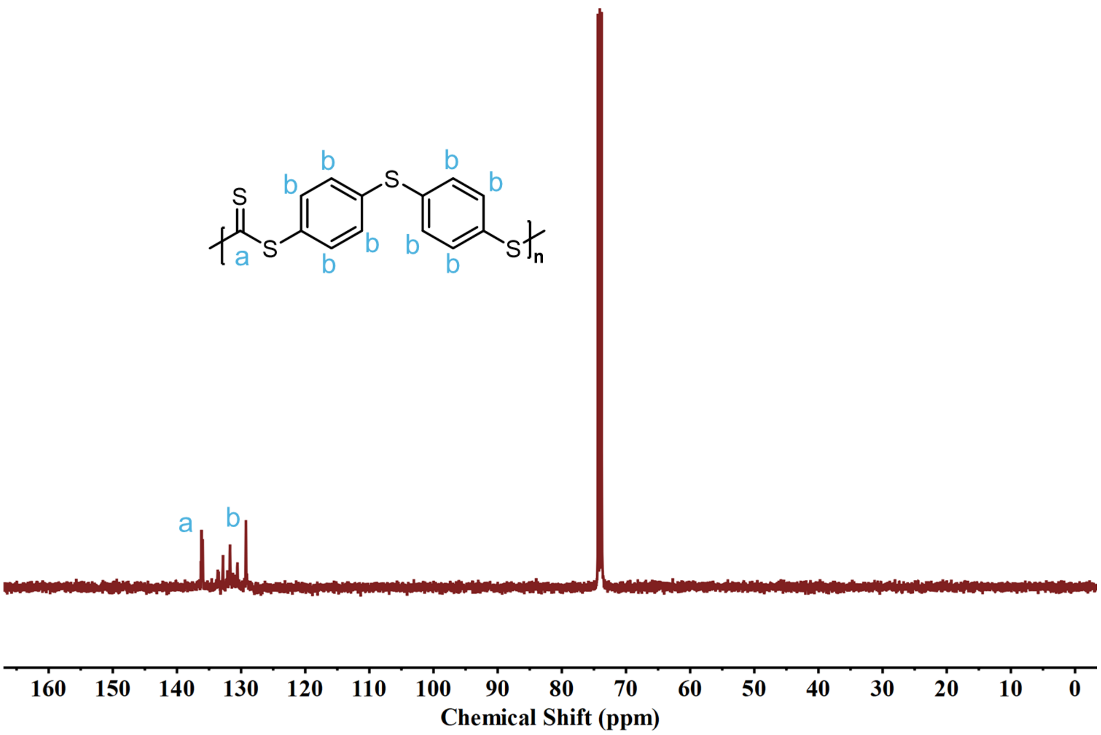

Supplementary Fig. 69 The ^{13}C NMR spectrum of **P11** at 120 °C in tetrachloroethane-d_2.

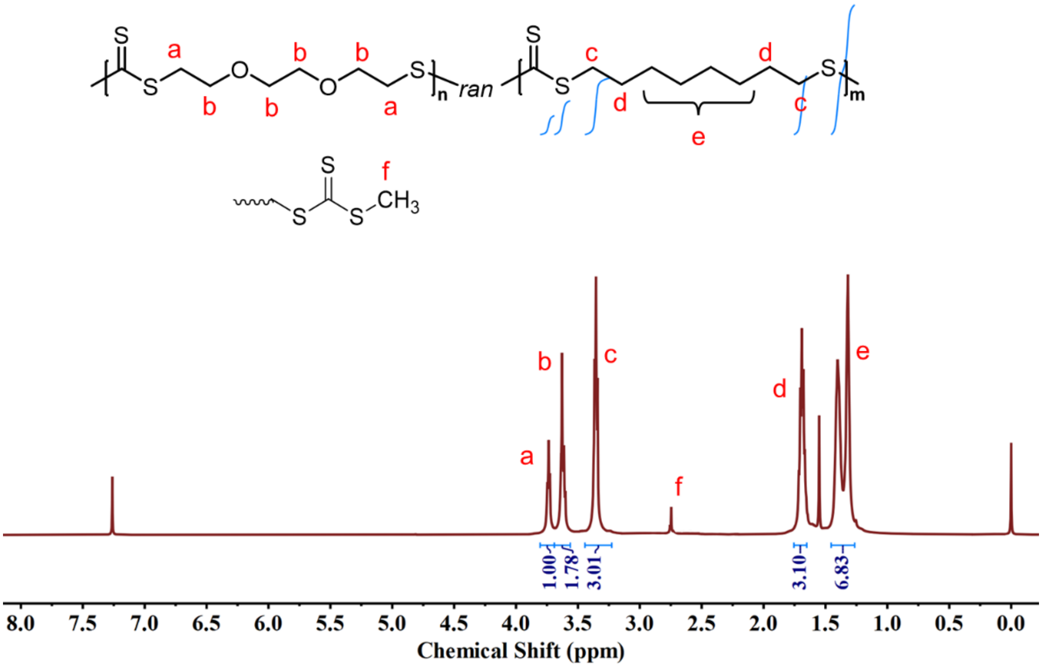

Supplementary Fig. 70 The ^{1}H NMR spectrum of **P12** at 40 °C in $CDCl_3$.

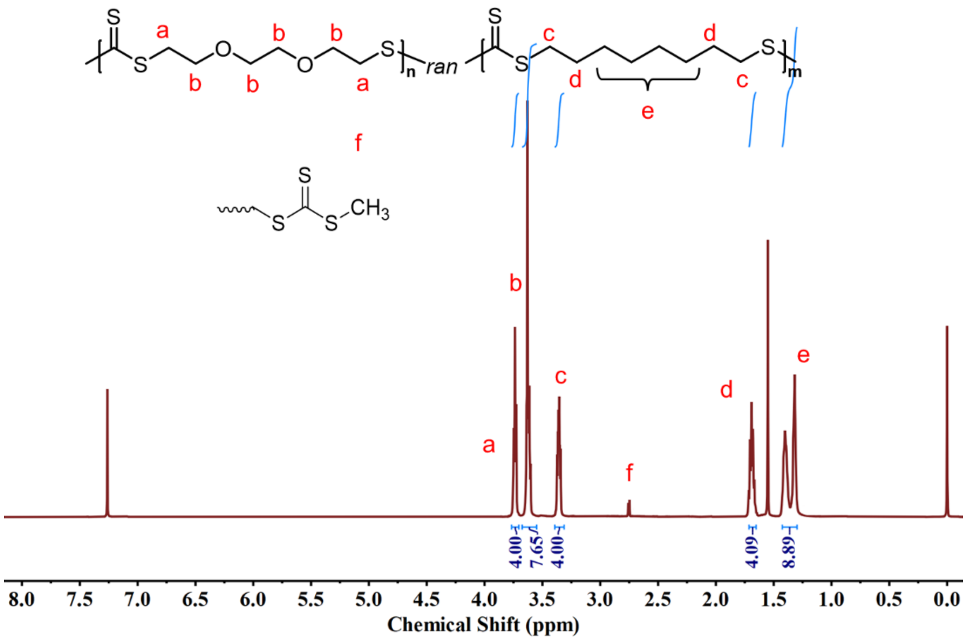

Supplementary Fig. 71 The ^{1}H NMR spectrum of **P13** at 40 °C in $CDCl_3$.

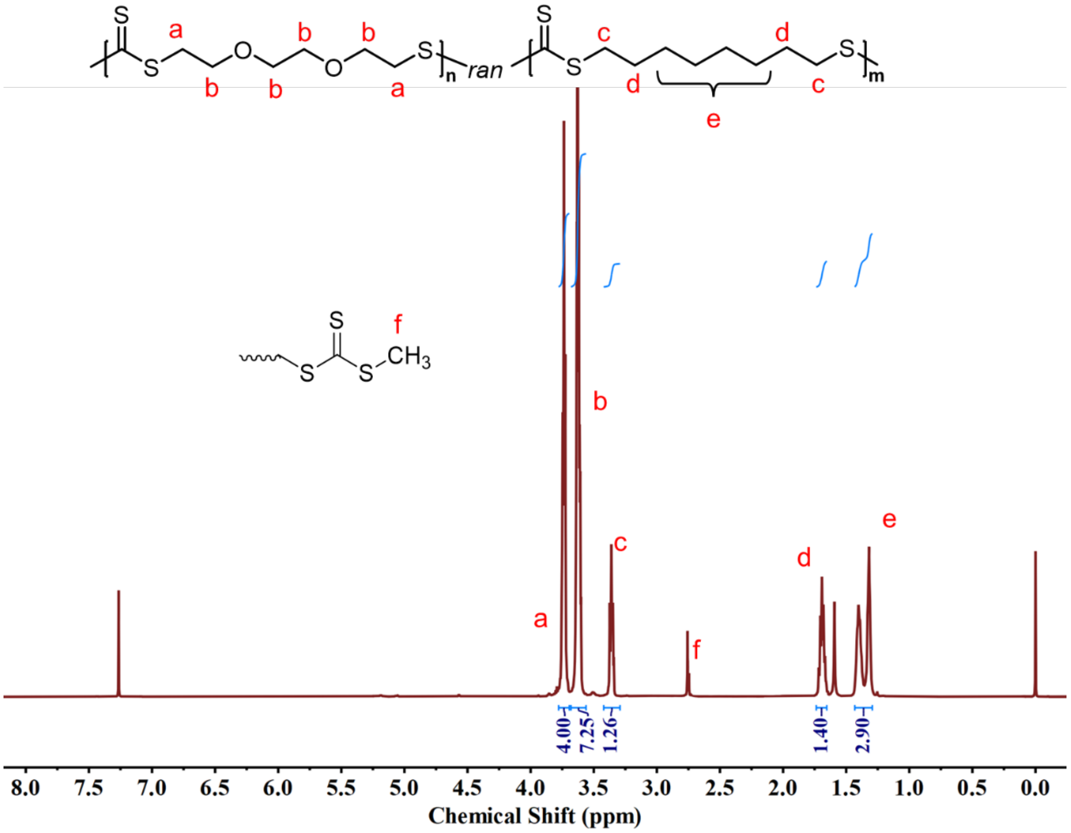

Supplementary Fig. 72 The ^{1}H NMR spectrum of **P14** at 25 °C in $CDCl_3$.

13. GPC traces of P1–P7

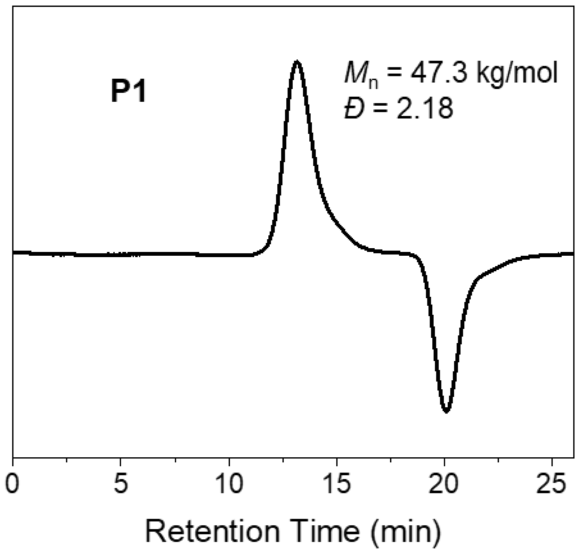

Supplementary Fig. 73 GPC trace of **P1**.

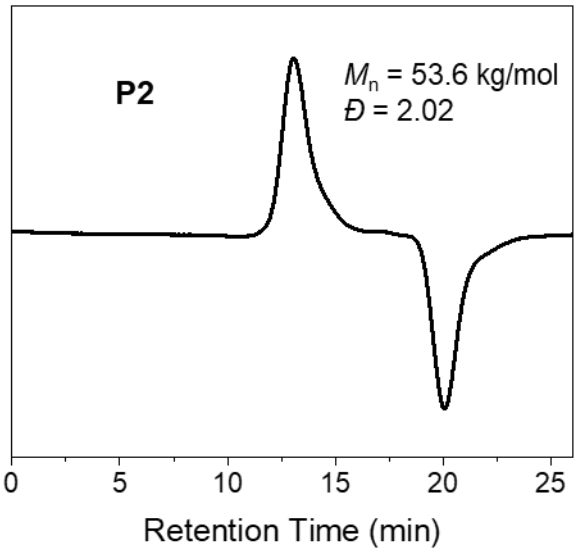

Supplementary Fig. 74 GPC trace of **P2**.

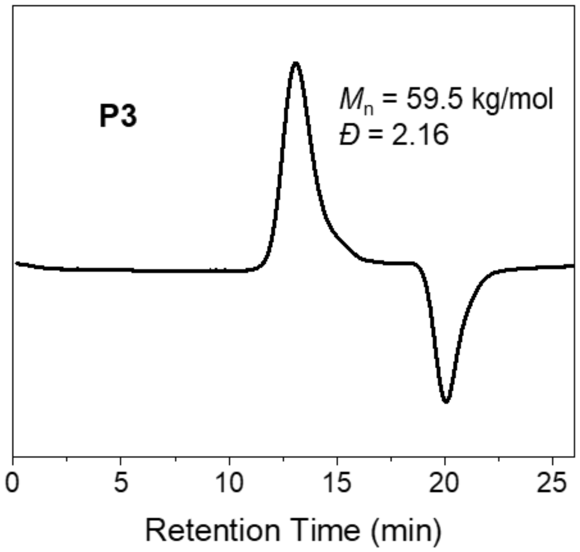

Supplementary Fig. 75 GPC trace of **P3**.

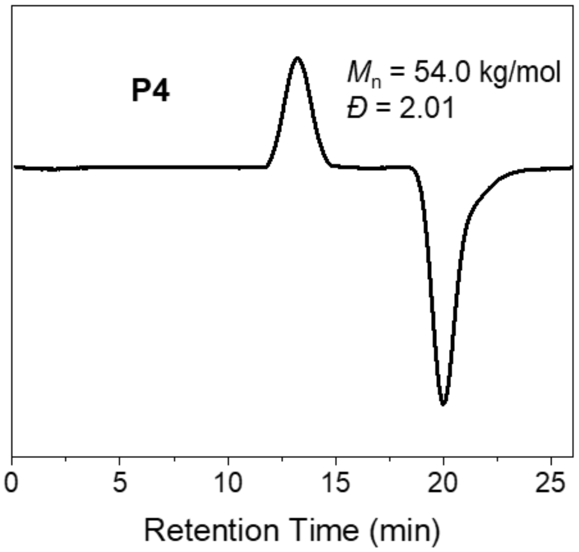

Supplementary Fig. 76 GPC trace of **P4**.

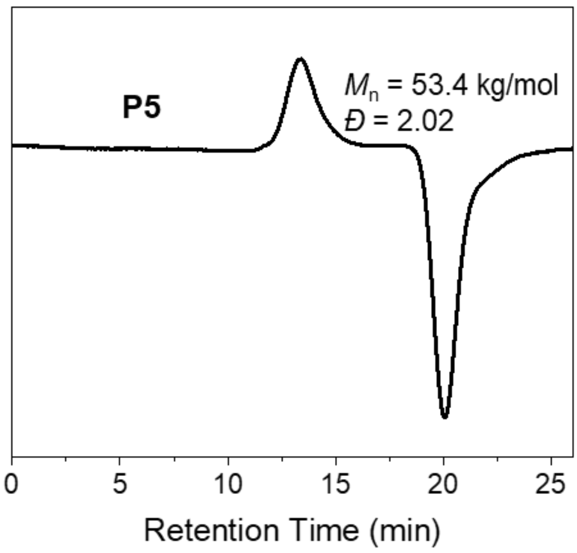

Supplementary Fig. 77 GPC trace of **P5**.

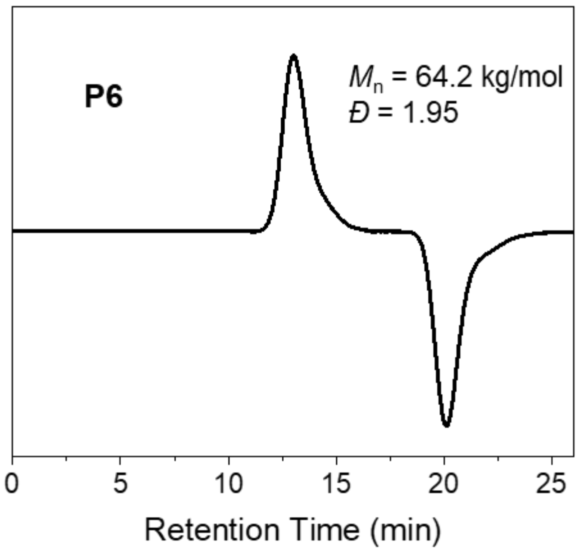

Supplementary Fig. 78 GPC trace of **P6**.

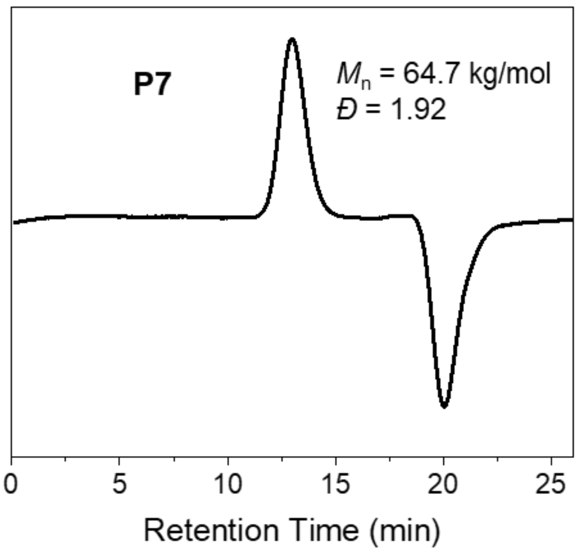

Supplementary Fig. 79 GPC trace of **P7**.

14.Reference

(1) Arzehgar, Z.; Ahmadi, H., A convenient one-pot method for the synthesis of symmetrical dialkyl trithiocarbonates using NH_4OAc under mild neutral conditions. *J. Chin. Chem. Soc.* **2019**, *66*, 303−306.

(2) SEK S. Two metal-molecule binding modes for peptide molecular junctions. *J. Phys. Chem. C* **2007**, 111, 12860−12865.
